# *Bacillus Licheniformis*‐Induced Cognitive Decline in Mice Linking Gut Microbiota Dysbiosis, Metabolic Perturbations, and Hippocampal Neuroinflammation

**DOI:** 10.1002/advs.78686

**Published:** 2026-11-09

**Authors:** Yibin Lin, Afeng Wang, Hongchang Zhou, Yuliang Sun, Yingying Guo, Keyu Chen, Junhong Sun, Tinliang Lin, Zhaochen Huang, Xinhua Qiang, Jie He, Jianlin Lou, Yunliang Yao, Shengwen Shao, Yongliang Pan, Xiang Wang

**Affiliations:** ^1^ School of Medicine Huzhou Normal University Huzhou China; ^2^ Key Laboratory for Precise Prevention and Control of Major Chronic Diseases Huzhou Normal University Huzhou China; ^3^ School of Science Huzhou Normal University Huzhou China; ^4^ School of Life Huzhou Normal University Huzhou China; ^5^ Clinical Laboratory First People's Hospital Affiliated to Huzhou Normal University Huzhou Normal University Huzhou China; ^6^ Infection Disease Department First People's Hospital Affiliated to Huzhou Normal University Huzhou Normal University Huzhou China; ^7^ Zhejiang Key Laboratory of Zero Magnetic Medicine School of Medicine Affiliated Hangzhou First People's Hospital Westlake University Hangzhou Zhejiang China

**Keywords:** cognitive function, gut microbiota, hippocampus, metabolome, neuroinflammation, probiotics, single‐cell RNA sequencing

## Abstract

While often considered beneficial, the long‐term effects of probiotic supplementation on brain function remain underexplored. This study demonstrates that prolonged oral administration of *Bacillus licheniformis* in mice induces significant cognitive decline, as evidenced by impaired social ability and social memory in the three‐chamber test. This behavioral deficit was associated with hippocampal structural abnormalities, including reduced neuronal soma size, dendritic length, and spine density. Multi‐omics analyses revealed that *B. licheniformis* disrupted gut microbiota homeostasis, driving specific shifts in microbial composition and function, notably enriching nucleotide metabolism pathways. Concomitant serum metabolomic profiling identified widespread perturbations, particularly in lipid, amino acid, and nucleotide metabolism. Single‐cell RNA sequencing of the hippocampus unveiled a remodeled cellular landscape, characterized by a marked increase in microglia and a decrease in oligodendrocytes. Transcriptomic analysis further indicated a shift in oligodendrocytes and astrocytes toward a reactive, pro‐inflammatory state, with significant activation of neuroinflammatory (e.g., NF‐κB) and neurodegenerative pathways. Our findings reveal an association between chronic *B. licheniformis* intake and cognitive impairment, suggesting a potential gut‐brain‐axis‐mediated pathogenic cascade involving intestinal dysbiosis, systemic metabolic disturbances, and hippocampal neuroinflammation. This work highlights the potential neurological risks of prolonged probiotic use and provides a framework for evaluating microbial impacts on brain health.

## Graphical abstract

Chronic oral administration of specific probiotic strains triggers gut microbiota dysbiosis and systemic metabolic perturbations in mice. Multi‐omics profiling reveals subsequent hippocampal glial remodeling and neuroinflammation, which are linked to cognitive impairment. This work highlights potential neurological risks from long‐term probiotic supplementation and underscores critical strain‐dependent safety considerations.

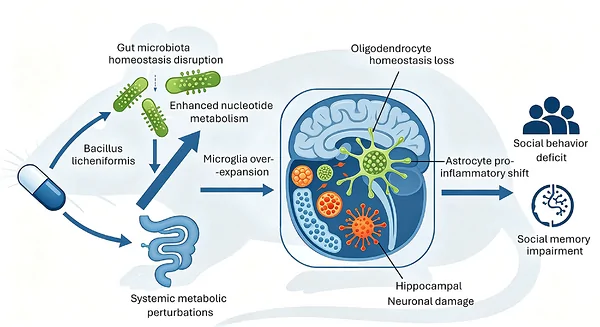

## 1 Introduction

The gut‐brain axis constitutes a complex, bidirectional communication network integrating neural, endocrine, immune, and metabolic pathways that link gastrointestinal function with central nervous system (CNS) activity [1]. Central to this axis is the gut microbiota, a vast and dynamic community of trillions of microbial cells that fundamentally influence host physiology, including systemic metabolism, immune homeostasis, and emotional and behavioral outputs [2]. Probiotics—live microorganisms conferring health benefits when administered in adequate amounts—are increasingly utilized to modulate this microbial milieu [3]. While traditionally investigated for gastrointestinal and immune‐modulating effects, emerging evidence indicates that specific strains, termed “psychobiotics”, can ameliorate symptoms of neuropsychiatric and neurodegenerative disorders [4, 5]. This expanding recognition has positioned probiotic supplementation as a mainstream strategy for cognitive health promotion [6, 7, 8, 9].

However, the long‐term neurological safety of chronic probiotic supplementation remains largely undefined. While existing human neurocognitive trials are predominantly limited to 4–12 weeks and utilize strain‐specific formulations, longer‐term safety data are restricted to narrow cohorts [10, 11, 12]. Furthermore, efficacy and tolerability are highly variable, depending on strain [11], dose [13], host status (e.g., stress or Mild Cognitive Impairment, MCI) [14], and intervention duration [15]. Strain identity dictates functional outcomes: while lactobacilli, bifidobacteria, and other psychobiotic strains typically confer neuroprotection via vagal or GABAergic signaling and anti‐inflammatory pathways [16, 17, 18, 19], chronic exposure to certain spore‐forming taxa (e.g., bacilli) has been implicated in disrupting gut homeostasis and triggering cognitive detriment [20, 21]. Dosage further modulates these effects; sustained high‐dose administration may displace indigenous beneficial taxa and enrich opportunistic commensals [22, 23]. Host heterogeneity, driven by genetics, baseline microbiota, and immune status, adds complexity, often outweighing chronological age as a predictive factor [24, 25, 26, 27, 28]. Crucially, duration acts as a pivotal determinant: short‐term supplementation (≤ 4 weeks) induces transient functional modulation [29, 30], whereas chronic exposure (≥ 12 weeks) drives sustained immunometabolic remodeling capable of durably altering cognitive phenotypes [31, 32]. This potential for maladaptive remodeling is underscored by preclinical data documenting persistent gut histological alterations and systemic inflammation following long‐term probiotic administration [23]. Collectively, these variables preclude the generalization of probiotics as universally beneficial, necessitating rigorous longitudinal assessment. Clinical case reports of “brain fog” and cognitive complaints following unsupervised, chronic probiotic use further highlight the potential for paradoxical neurotoxicity in susceptible hosts [33, 34].

This raises a critical, yet underexplored, question: Could long‐term ingestion of specific probiotic strains—intended to confer health benefits—paradoxically precipitate cognitive decline? The gut microbiome functions as a complex ecological network; introducing exogenous microbial strains can perturb microbial composition, metabolic output, and immune signaling in unforeseen ways [6, 35]. Such perturbations may propagate to the CNS via the gut–brain axis, potentially exacerbating neuroinflammation, disrupting neurotransmitter homeostasis, and impairing synaptic plasticity [36]. Consequently, well‐intentioned probiotic regimens might, under conditions of prolonged use or in susceptible individuals, inadvertently drive neurodegeneration. This possibility remains largely unexamined despite the global prevalence of chronic, unregulated probiotic consumption [37]. Recent evidence emphasizes that probiotic outcomes are highly strain‐specific and context‐dependent, suggesting that some microbial interventions may disrupt host–microbe homeostasis with unintended neurological sequelae [38]. Addressing this knowledge gap is imperative, particularly for vulnerable populations such as the elderly or individuals with pre‐existing inflammatory conditions.

To investigate this hypothesis, we postulated that chronic supplementation with specific probiotic strains—particularly spore‐forming taxa—can paradoxically impair cognitive function through gut dysbiosis and hippocampal neuroinflammation. To test this, we administered five phylogenetically distinct, generally recognized as safe (GRAS) probiotic strains (*Bacillus licheniformis*, *Clostridium butyricum*, *Bacillus cereus*, *Lactobacillus acidophilus*, and *Bifidobacterium longum* subsp. *longum*) to mice via oral gavage for six months (Figure 1). These strains spanned both spore‐forming and non‐spore‐forming taxa, representing a broad spectrum of commercially relevant probiotics. Integrated behavioral and histomorphological assessments revealed that *B. licheniformis* uniquely induced the most severe cognitive deficits and hippocampal architectural abnormalities. In stark contrast, the other strains elicited either modest neuroprotective effects (e.g., *L. acidophilus*) or no significant behavioral alteration, prompting our exclusive focus on *B. licheniformis* for deep mechanistic interrogation.

**FIGURE 1:**
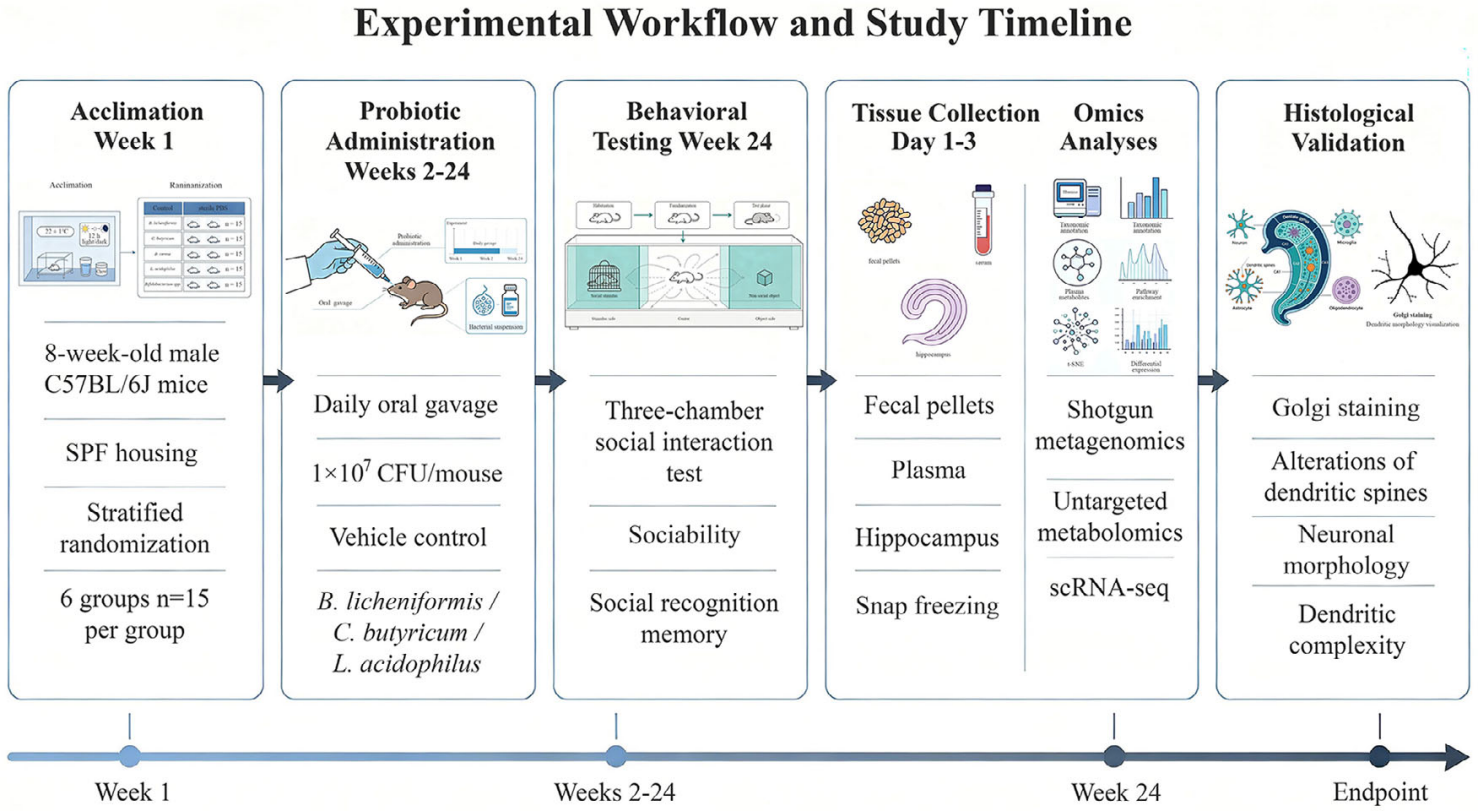
Experimental workflow and study timeline. Ninety male C57BL/6J mice were randomized into six groups (n = 15 per group: Control, *B. licheniformis*, *C. butyricum*, *B. cereus*, *L. acidophilus*, and *B. longum* subsp. *longum*) and received daily oral gavage with vehicle (PBS) or one of five probiotic strains for six months. Cognitive function was assessed via the three‐chamber test during the final week. Fecal pellets, serum, and hippocampal tissues were collected for shotgun metagenomic sequencing, untargeted metabolomics (LC‐MS/MS), single‐cell RNA sequencing (10x Genomics), and histomorphological analysis (Golgi staining). Integrated multi‐omics analysis revealed the mechanistic cascade linking gut microbiota dysbiosis to systemic metabolic perturbation, hippocampal neuroinflammation, and cognitive decline. Fecal and hippocampal tissues were pooled per experimental group for metagenomic and single‐cell RNA‐seq library preparation; serum metabolomics, behavioral, and histological assessments were performed on samples from individual non‐pooled mice.

We subsequently employed a multi‐omics approach to elucidate the mechanistic underpinnings of this observation. Cognitive performance and hippocampal neuronal morphology were rigorously assessed alongside shotgun metagenomic sequencing of the gut microbiota, untargeted serum metabolomics, and single‐cell RNA sequencing (scRNA‐seq) of hippocampal tissues. Our integrated multi‐omics analyses reveal a coherent associative pathogenic cascade correlating chronic *B. licheniformis* exposure with cognitive impairment. These observations point to a potential gut‐brain‐mediated sequence of events spanning intestinal dysbiosis, systemic metabolic perturbation, and hippocampal neuroinflammation. Definitive causal validation would require dedicated functional experiments such as fecal microbiota transplantation or mono‐colonization assays using germ‐free mice. These findings challenge the prevailing assumption of universal probiotic safety and underscore the critical necessity for strain‐specific, long‐term evaluations of probiotic interventions targeting the gut–brain axis.

## 2 Results

### 2.1 Probiotic Intervention Causes a Significant Decline in Cognitive Function in Mice

To evaluate the potential impact of prolonged probiotic supplementation on cognitive function, we orally administered five common probiotic strains—*B. licheniformis*, *B. cereus*, *C. butyricum*, *B. longum* subsp. *longum*, and *L. acidophilus*—to specific pathogen‐free (SPF) C57BL/6 mice via daily gavage for six months. Cognitive performance was assessed using the three‐chamber test for social interaction and social memory (Figure 2a). Mice receiving *B. cereus* or *B. longum* subsp. *longum* died before the six‐month endpoint, precluding cognitive evaluation in these groups.

**FIGURE 2:**
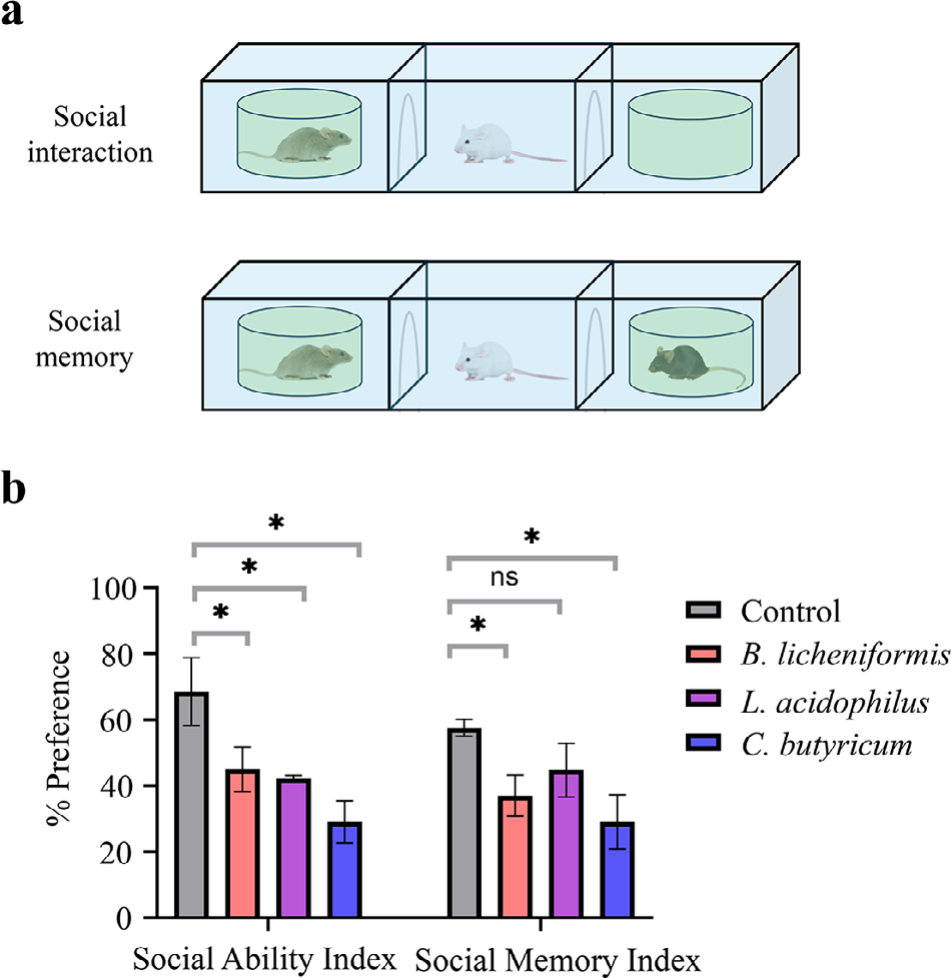
Long‐term probiotic administration impairs cognitive function. (a) Schematic of the three‐chamber test for assessing social interaction and social memory. In the social interaction test, test mice are allowed to explore an arena containing an empty cylinder, a novel conspecific mouse, and another empty cylinder. In the social memory test, conducted 24 h later, one of the empty cylinders now contains a familiar mouse, testing the mouse's ability to distinguish between novel and familiar individuals. (b) Results of social ability and social memory tests in control and probiotic‐treated mice (*B. licheniformis*, *L. acidophilus*, *C. butyricum*). The bar graph shows the percentage preference index for sociability (left panel) and social memory (right panel). Data are presented as mean ± SEM. *n* = 6 mice per group (Control, *B. licheniformis*, *L. acidophilus*, and *C. butyricum*). Statistical significance was assessed using one‐way ANOVA followed by Tukey's post‐hoc test: ns (not significant), *P < 0.05, **P < 0.01, ***P < 0.001.

Figure 2b illustrates the effects of the three remaining probiotics—*B. licheniformis*, *L. acidophilus*, and *C. butyricum*—on social ability and social memory, expressed as percentage preference in the three‐chamber test. The control group (gray) showed the highest performance in both measures (social ability: 68.59% ± 10.30%; social memory: 57.63% ± 2.56%). Among probiotic‐treated groups, *B. licheniformis* yielded moderate scores (social ability: 44.99% ± 6.80%; social memory: 36.98% ± 6.22%). *L. acidophilus* resulted in lower social ability (42.39% ± 0.74%) but higher social memory (44.76% ± 8.14%), whereas *C. butyricum* produced the lowest scores (social ability: 29.08% ± 6.39%; social memory: 29.06% ± 8.20%). In social ability, all three probiotic groups showed a significant decrease in percent preference compared with the control (*p* < 0.05). In social memory, *B. licheniformis* and *C. butyricum* also caused a significant reduction (*p* < 0.05), while the *L. acidophilus* group did not differ significantly from the control (*p*  =  0.105), suggesting minimal impact on social memory under the experimental conditions.

To explore potential neurostructural correlates of these cognitive deficits, we performed Golgi staining on hippocampal sections from the surviving probiotic‐treated mice. Golgi staining revealed marked morphological alterations in neurons from all three probiotic groups (Figure 3). Soma area was significantly reduced across all treatments compared with the control, which showed broader distribution and larger average cell‐body size (Figure 3a,b). Total dendritic length was also shorter in all probiotic groups relative to controls, with *C. butyricum* exhibiting moderately longer dendrites than *B. licheniformis* and *L. acidophilus* (Figure 3c). Dendritic spine density (per 10 µm) was considerably lower in all experimental groups, especially in *C. butyricum*‐treated mice, which displayed the fewest spines (Figure 3d,e). Sholl analysis indicated reduced neuronal complexity, with fewer dendritic intersections in treated groups, particularly within 20 µm of the soma. *L. acidophilus* and *B. licheniformis* groups showed statistically significant decreases in intersection numbers at specific proximal distances compared with controls (Figure 3f). Overall, probiotic administration consistently reduced neuronal soma size, dendritic length, spine density, and arborization complexity.

**FIGURE 3:**
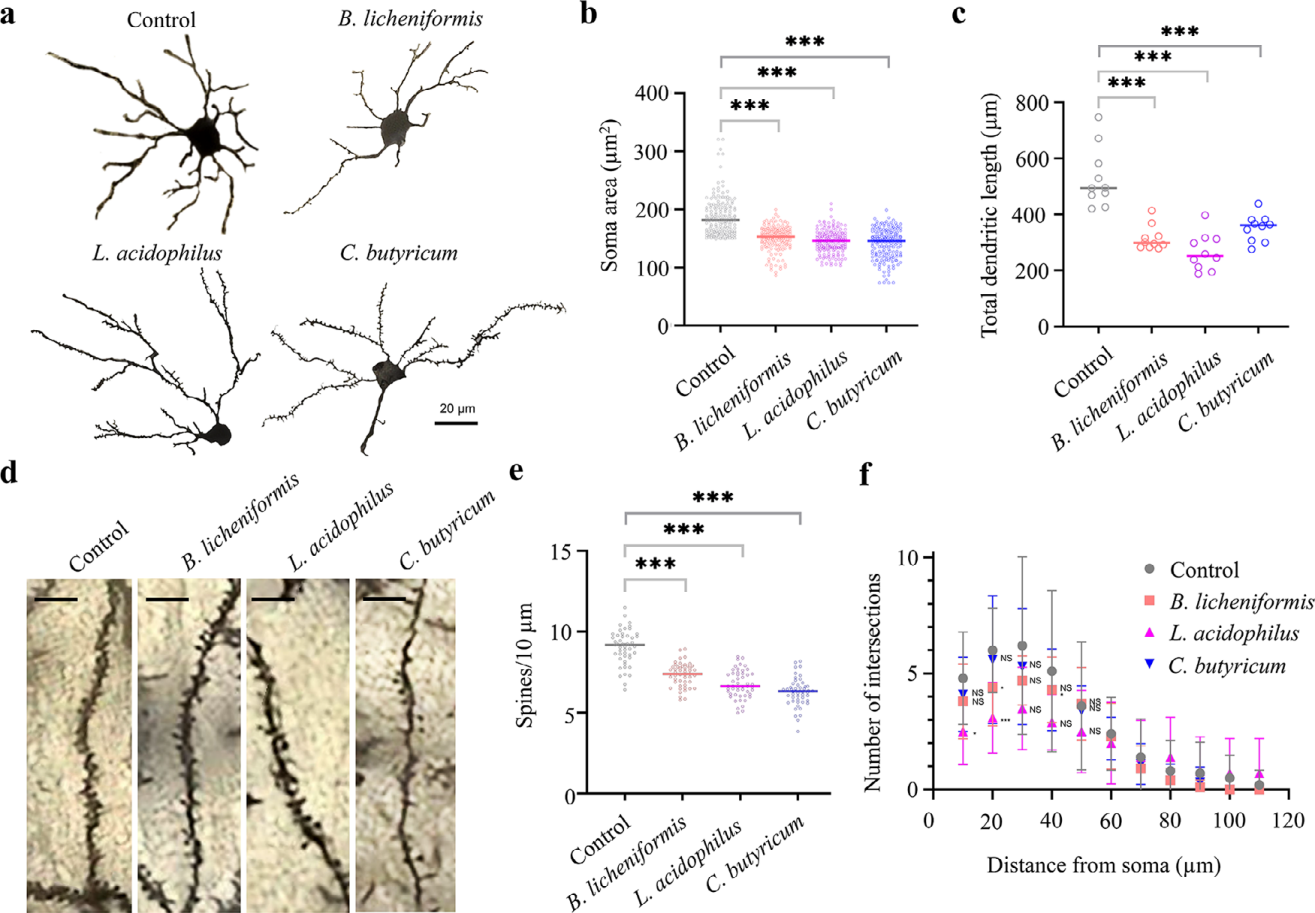
Long‐term probiotic administration induces hippocampal neuronal structural abnormalities. (a) Representative Golgi‐stained images showing neuronal morphology in the hippocampus from the Control, *B. licheniformis*, *L. acidophilus*, and *C. butyricum* groups. (b) Quantification of neuronal soma area. (c) Measurement of total dendritic length. (d) Representative dendritic spine images and a schematic illustrating spine distribution along the dendrites. Scale bar = 10 µm. (e) Quantitative assessment of dendritic spine density (spines/10 µm). (f) Sholl analysis of neuronal intersections at varying distances from the soma. Data are presented as mean ± SEM. *n* = 6 mice per group (Control, *B. licheniformis*, *L. acidophilus*, and *C. butyricum*). ns (not significant), *p < 0.05, **p < 0.01, ***p < 0.001 vs. control group.

Together, these behavioral and histomorphological findings demonstrate that long‐term administration of specific probiotics induces significant cognitive impairment and is accompanied by structural abnormalities in the hippocampus, suggesting adverse effects on synaptic organization and neuronal health.

### 2.2 Probiotics Disrupt the Homeostasis of Gut Microbiota, Inducing Dysbiosis

Increasing evidence has indicated the role of gut microbiota in the progression of cognitive impairment; thus, we analyzed the composition, abundance, and function of gut microbiota with fecal samples in probiotic‐administered mice via metagenome sequencing. In this experiment, mice were administered three probiotics—*B. licheniformis*, *L. acidophilus*, and *C. butyricum*—with a water‐administered group serving as the control. Fecal samples collected from probiotic‐administered groups and control group were subsequently subjected to metagenome shotgun sequencing on an Illumina HiSeq platform to investigate the specific regulatory effects of different probiotics on the gut microbiota structure of mice (Tables S1 and S2, and Figure S1). The high‐quality sequencing reads were aligned to a mouse‐specific gut microbiome reference, MRGM (Mouse Reference Gut Microbiome) [39], which enabled high mapping rates and good coverage of the mouse gut microbiome for downstream analyzes. Analyses were conducted at the phylum, genus, and species levels, combined with multivariate statistical approaches.

After interventions with the three probiotics—*B. licheniformis*, *C. butyricum*, and *L. acidophilus*—the structure of the intestinal microbial community in mice changed significantly at the phylum level (Figure 4a, Table S3, Figures S1 and S2). Among them, the treatment with *C. butyricum* induced the most pronounced changes. The abundance of Firmicutes decreased to 31.35%, lower than that of the control group (39.30%), while the abundance of Bacteroidetes increased substantially to 51.00%, far exceeding that of the control group (25.82%). This resulted in a fundamental reversal of the ratio between the two phyla. At the same time, the abundance of Chlamydiae decreased to 7.66% and 9.75% in the *B. licheniformis* and *C. butyricum* groups, respectively, which was significantly lower than that in the control group (29.55%) and the *L. acidophilus* group (23.28%). In contrast, the abundance of Proteobacteria increased to 9.67% in the *B. licheniformis* group, higher than that in the control group (2.80%). These results suggest that probiotic intervention can reshape the overall structure of the gut microbiota. The fundamental changes in the proportions of core phyla and the fluctuations in phyla associated with conditional pathogens (such as Proteobacteria) may represent potential microbiological features accompanying cognitive decline after intervention, as the structure of the gut microbiota is closely linked to the health of the host's nervous system.

**FIGURE 4:**
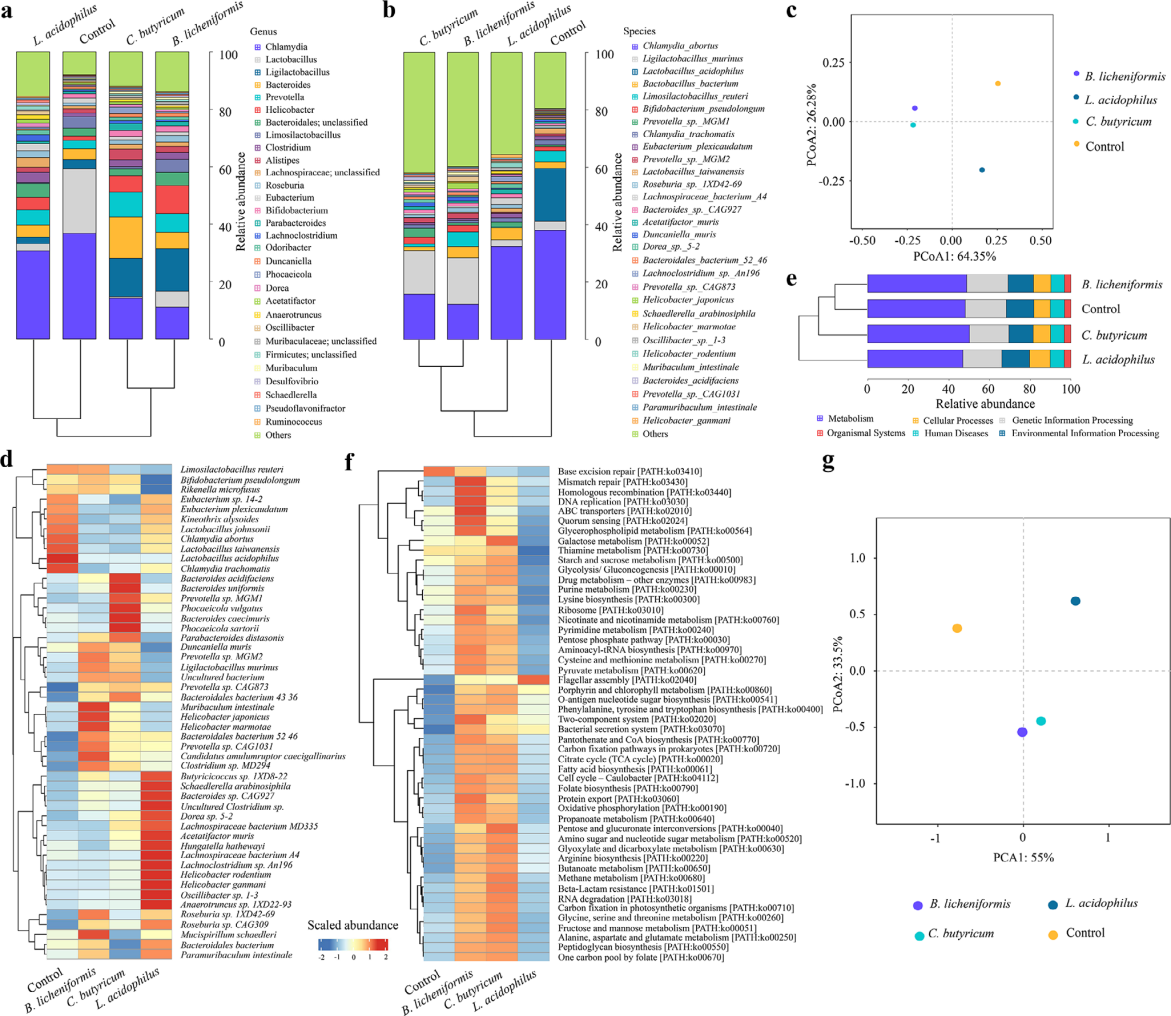
Alterations in gut microbiota composition of mice after probiotic intervention. (a) Community composition at the genus level, presented as a stacked bar chart across the Control, *B. licheniformis*, *L. acidophilus*, and *C. butyricum* groups. (b) Species‐level taxonomic profiles illustrated by stacked bar plots. (c) Principal Coordinate Analysis (PCoA) of species composition, which reveals distinct clustering of microbial communities among the different groups (PCoA1: 64.35%, PCoA2: 26.28%). (d) Clustered heatmap depicting the relative abundance of the most prevalent bacterial species. (e) Predicted functional capacity of the gut microbiota based on KEGG pathways, categorized into six major modules: Metabolism, Organismal Systems, Cellular Processes, Human Diseases, Genetic Information Processing, and Environmental Information Processing. (f) Heatmap representation of KEGG pathway abundance, highlighting specific metabolic and signaling cascades. (g) Principal Component Analysis (PCA) score plot derived from species abundance data, explaining 55% and 33.5% of the total variance on PC1 and PC2, respectively. Fecal pellets from 10 mice per group were pooled to generate one single metagenomic sequencing library for each experimental group.

At the species level, these specific alterations became clearer and more detailed (Figure 4b, Table S4, and Figure S3). The intestinal microbiota composition in all probiotic treatment groups showed significant alterations compared to the control group, with a notably increased proportion of the “others” category, which may reflect shifts in low‐abundance taxa rather than necessarily indicating greater overall diversity. Appropriate alpha‐ and beta‐diversity‐based ordination analyses (e.g., PCoA in Figure 4c) revealed distinct community‐level clustering patterns between experimental groups, noting these profiles originate from pooled fecal samples without biological replicate sequencing. Specific species analysis revealed that the abundance of *Ligilactobacillus murinus* increased substantially in the *B. licheniformis* group but decreased in the *L. acidophilus* group, while *Lactobacillus acidophilus* was extremely low in its namesake treatment group and highest in the control group, indicating that exogenous supplementation did not colonize and may even suppress endogenous strains (Table S4 and Figure S3). Meanwhile, the abundance of *Lactobacillus taiwanensis* decreased in the *C. butyricum* group, and opportunistic pathogens such as *Chlamydia abortus* were elevated in the *B. licheniformis* group, with *Helicobacter* species showing an increase in *L. acidophilus* treatment groups.

Results from the species abundance heat map further confirmed the above findings (Figure 4d). Cluster analysis intuitively grouped bacterial species with similar response patterns to the different treatments, clearly demonstrating the specific microbiota enrichment pattern corresponding to each probiotic. Principal Coordinates Analysis (PCoA), from the perspective of overall community β‐diversity, strongly supported the existence of these specific alterations (Figure 4c). The PCoA results showed that pooled‐sample‐derived profiles from the four groups formed distinct clustering patterns in 2D space. The *B. licheniformis* and *C. butyricum* groups were relatively close to each other but distant from both the *L. acidophilus* group and the control group. Together, these results indicate that all three probiotic treatments significantly drove restructuring of the gut microbial community, with each probiotic guiding the community succession in a unique direction, thereby producing specific probiotic effects.

Based on the results of the metagenomic analysis, the three probiotic treatments induced significant and specific alterations in the functional profile of the mouse gut microbiota. Kyoto Encyclopedia of Genes and Genomes (KEGG) functional relative abundance cluster analysis revealed differences in the distribution across six major functional categories between the probiotic treatment groups and the control group (Figure 4e). Metabolic functions showed the highest relative abundance in the control group (48%), with slight decreases to 42%, 40%, and 40% in the *B. licheniformis*, *C. butyricum*, and *L. acidophilus* treatment groups, respectively, suggesting that probiotic intervention may have mildly suppressed overall metabolic activity. Other categories, such as genetic information processing and environmental information processing, exhibited fluctuations in the probiotic groups. However, dendrogram clustering based on Bray distance revealed that the functional composition of the *B. licheniformis* treatment group was more closely related to the control group, whereas the *L. acidophilus* and *C. butyricum* groups clustered together, indicating that these two strains may share some functional characteristics. The heatmap in Figure 4f further detailed changes at the KEGG level 3 pathway level. The control group showed higher abundance in carbohydrate metabolism pathways such as glycolysis/gluconeogenesis, reflecting active basal energy metabolism. The *B. licheniformis* treatment group was significantly enriched in nucleotide metabolism‐related pathways, such as pyrimidine metabolism and base excision repair, highlighted in red, suggesting that this strain may promote DNA repair and replication processes. The *L. acidophilus* and *C. butyricum* treatment groups both activated amino acid biosynthesis pathways (e.g., lysine and arginine metabolism) and membrane transport systems (e.g., ABC transporters). However, heatmap clustering indicated that while there was overlap between the two, the *L. acidophilus* group was more prominent in amino acid synthesis, whereas the *C. butyricum* group showed greater enrichment in transport pathways, reflecting strain‐specific regulation. Principal Component Analysis (PCA) analysis in Figure 4g integrated these functional differences. PCA1 (55% variance) and PCA2 (33.5% variance) clearly separated the groups. The *L. acidophilus* treatment group was located in the positive direction of PCA1 and exhibited the greatest distance from the control group, indicating the most significant functional changes, potentially related to enhanced amino acid metabolism. The *C. butyricum* group was also in the positive direction of PCA1 but symmetrically distributed relative to *L. acidophilus* along the PCA2 axis, suggesting a different regulatory focus, possibly emphasizing membrane transport functions. The *B. licheniformis* group deviated from the control but was relatively closer, indicating milder changes, consistent with its enrichment in nucleotide metabolism.

Of note, both shotgun metagenomic and hippocampal scRNA‐seq datasets were generated from group‐pooled biological materials, yielding only one aggregated sequencing profile per experimental group. This sample‐pooling design precludes individual‐animal‐based correlation or multi‐omics network analyses, which typically require 3–5 independent biological replicates per group at the individual‐mouse level. Importantly, untargeted serum metabolomics, behavioral testing, and histological assessments were performed on non‐pooled samples derived from individual mice with biological replicates (n = 15 per group). In the present work, congruent group‐level alterations across microbiome, metabolome, hippocampal transcriptome, histomorphology, and neurobehavioral phenotypes are interpreted as convergent observational evidence supporting probiotic‐linked gut‐brain‐axis disturbance. Given this pooling design, formal statistical evaluation of within‐group microbial variation and replicate‐based differential‐abundance analysis (such as LEfSe) cannot be applied to the shotgun metagenomic dataset.

### 2.3 Blood Metabolomics Reveals Microbiota‐Metabolic Disorder

Serum samples were analyzed by untargeted liquid chromatography/mass spectrometry (LC/MS). After quality control, data filtering, and normalization (see Methods), we identified 57,576 metabolic features across 9 samples (Table S5). Of these, 1521 secondary qualitative substances were retained (Figure 5a and Figure S4).

**FIGURE 5:**
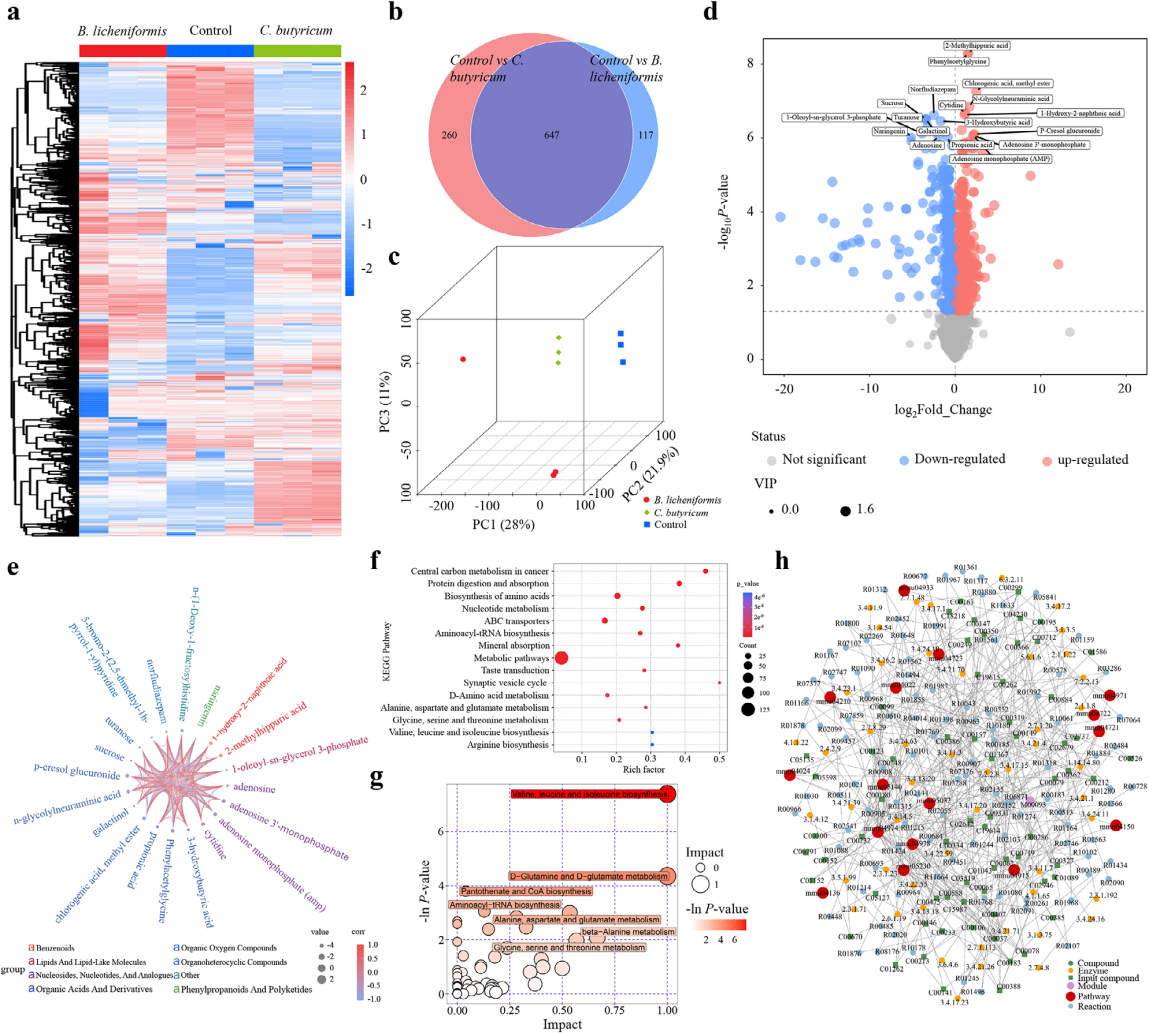
Altered serum metabolites in mice following probiotic intervention compared to control. (a) A heatmap depicting the results of hierarchical clustering analysis for all samples, displaying the scaled abundance of metabolites in the Control, *B. licheniformis*, and *C. butyricum* groups. (b) A Venn diagram illustrates the overlap of differentially abundant metabolites from the Control vs. *C. butyricum* and Control vs. *B. licheniformis* comparisons, indicating 260 unique metabolites in the former, 647 shared, and 117 unique to the latter. (c) Three‐dimensional principal component analysis (PCA) score plot of the three groups, demonstrating clear metabolic separation (PC1: 28%, PC2: 21.9%, PC3: 11%). (d) Volcano plot displaying the log_2_(fold change) versus ‐log_10_(*P*‐value) for the *B. licheniformis* versus control comparison. Red dots denote significantly upregulated metabolites, blue dots indicate downregulated ones, and gray dots represent non‐significant metabolites (adjusted *P*‐value ≥ 0.05). (e) Chord diagram mapping the classification and correlations of altered metabolites in the *B. licheniformis* group relative to controls. The outer ring lists specific metabolite names (e.g., sucrose, turanose, adenosine, chlorogenic acid methyl ester), with inner ribbons connecting them to their respective chemical classes. These metabolites are grouped into categories such as Benzenoids; Lipids and Lipid‐Like Molecules; Nucleosides, Nucleotides, and Analogues; Organic Acids and Derivatives; Organic Oxygen Compounds; Organoheterocyclic Compounds; Phenylpropanoids and Polyketides; and Others. Colors correspond to these classifications as shown in the legend. (f) Bubble plot of KEGG pathway enrichment analysis for the differentially abundant metabolites. Dot size is proportional to the number of annotated metabolites, and the color gradient reflects the *P*‐value. (g) Pathway impact bubble plot summarizing the enrichment results. The x‐axis indicates the pathway impact value, and the y‐axis shows the ‐ln(*P*‐value). Bubble size represents the impact magnitude, and the color intensity (red gradient) denotes the ‐ln(*P*‐value), with deeper red signifying greater significance. Highlighted pathways include valine, leucine, and isoleucine biosynthesis; D‐glutamine and D‐glutamate metabolism; pantothenate and CoA biosynthesis; aminoacyl‐tRNA biosynthesis; alanine, aspartate, and glutamate metabolism; beta‐alanine metabolism; and glycine, serine, and threonine metabolism. (h) Regulatory network analysis of the key differential metabolites, illustrating the complex interactions among compounds, enzymes, and metabolic pathways. Nodes are color‐coded and shaped by molecular classification: green squares for compounds, yellow hexagons for enzymes, orange inverted triangles for lipid compounds, red circles for pathways, and purple circles for modules. Blue lines represent reactions. Node size is proportional to the variable importance in projection (VIP) value or count, while color intensity reflects the magnitude of the ‐ln(*P*‐value). *n* = 3 mice per group (Control, *B. licheniformis*, and *C. butyricum*), with 9 samples in total.

Metabolomic analysis confirmed that both *C. butyricum* and *B. licheniformis* treatments significantly affected the host metabolic state. As shown in the Venn diagram (Figure 5b), there were distinct intergroup differences in the differential metabolites in mouse blood induced by the two treatments. In the Control vs *C. butyricum* comparison, 907 differential metabolites were identified, of which 260 were specific to *C. butyricum*; in the Control vs *B. licheniformis* comparison, 764 differential metabolites were found, with 117 specific to *B. licheniformis*. Notably, 647 differential metabolites were shared between the two comparisons, indicating that while both treatments induced widespread metabolic changes, they also affected a large number of common metabolic pathways, suggesting partial overlap in their mechanisms of action. The 3D PCA plot (Figure 5c) further revealed that sample points from the control group, *C. butyricum*‐treated group, and *B. licheniformis*‐treated group clustered separately in principal component space, demonstrating that each treatment exerted significant and reproducible effects on the overall metabolic profile.

The volcano plot (Figure 5d) and chord diagram (Figure 5e) reveals that, compared with the control group, the serum metabolic profile of mice in the *B. licheniformis* intervention group underwent extensive and profound remodeling, with a large number of significantly up‐regulated and down‐regulated differential metabolites. The significantly down‐regulated metabolites included substances that may affect sedation‐wakefulness balance, such as norfludiazepam; energy‐supplying carbohydrates for the brain, such as 3‐hydroxybutyric acid and sucrose; the neuroprotective compound naringenin; and signaling molecules like 1‐oleoyl‐sn‐glycero‐3‐phosphate. Concurrently, significantly up‐regulated metabolites included gut microbiota‐derived metabolites such as 2‐methylhippuric acid, phenylacetyl glycine, and p‐cresol glucuronide, whose accumulation may be associated with potential neurotoxicity. Additionally, the elevation of purine metabolites like adenosine 3'‐monophosphate also suggests potential energetic stress or pathway abnormalities. These changes collectively indicate that the probiotic may exert a negative impact on cognitive function through multiple interrelated pathways: a reduction in neuroprotective substances and a potential enhancement of neural inhibitory signaling may impair neuronal function and alertness; the widespread down‐regulation of energy substrates suggests that peripheral energy supply to the brain may be restricted; and the abnormal accumulation of various gut microbiota‐derived metabolites strongly indicates that altered metabolic communication via the “gut‐brain axis” may play a central role in mediating the adverse central effects.

Following the administration of *C. butyricum*, untargeted serum metabolomic analysis revealed significant alterations in the serum metabolic profile of mice, indicating that the intervention substantially perturbed the host's systemic metabolic state (Figure S5a,b). These changes exhibited a specific disruption pattern, potentially linked to the observed cognitive decline phenotype. Specifically, metabolites with neuroprotective or energy‐supporting functions were generally reduced. For instance, the anti‐inflammatory and neuroprotective compound luteolin‐7‐glucoside, the neuromodulator adenosine, the alternative brain energy substrate 3‐hydroxybutyric acid, and tetradecenoyl carnitine (which may indicate compromised mitochondrial function) were all significantly down‐regulated. In parallel, levels of multiple substances with potential neurotoxicity or pro‐inflammatory properties were elevated. Notably, gut microbiota‐derived uremic toxins, p‐cresol sulfate and phenyl sulfate, which can cross the blood‐brain barrier and induce oxidative stress and neuroinflammation, were significantly up‐regulated. An increase in the essential amino acid methionine might also elevate cognitive impairment risk via associated metabolic pathways. In summary, the *B. licheniformis* intervention induced an imbalance between “pro‐inflammatory/pro‐injury” and “neuroprotective/energy‐supportive” signals within the serum metabolic profile. This specific metabolic disruption pattern likely contributes to cognitive decline by exacerbating neuroinflammation and affecting cerebral energy metabolism and mitochondrial function, providing crucial clues for elucidating the potential metabolic mechanisms by which this probiotic impacts cognition.

Untargeted metabolomic analysis of serum from mice fed *B. licheniformis* and subsequent pathway enrichment analysis of differential metabolites using the KEGG database revealed that the intervention induced extensive alterations in serum metabolites, with significant enrichment in multiple metabolic pathways (Figure 5f,g). Among these, pathways including central carbon metabolism in cancer, protein digestion and absorption, amino acid biosynthesis, and nucleotide metabolism were highly enriched, suggesting that *B. licheniformis* may indirectly affect neural function by modulating energy and fundamental metabolism. Crucially, several pathways potentially associated with cognitive function were also significantly enriched: disruption of synaptic vesicle cycle suggests potential impairment in neurotransmitter storage and release processes. Furthermore, alterations in a series of amino acid metabolism pathways—such as alanine, aspartate and glutamate metabolism; glycine, serine and threonine metabolism; valine, leucine and isoleucine biosynthesis; and arginine biosynthesis—may collectively impact the balance of glutamate (a major excitatory neurotransmitter), NMDA receptor function, brain energy supply, and nitric oxide‐mediated synaptic transmission. Additionally, changes in pathways like D‐amino acid metabolism and ABC transporters may directly or indirectly influence synaptic transmission and the brain's microenvironment. These results collectively indicate that *B. licheniformis* may affect the cognitive function of mice by broadly disrupting amino acid metabolic balance, synaptic vesicle cycling, and neurotransmitter‐related metabolism, thereby providing directions for subsequent mechanistic validation targeting specific pathways.

Based on KEGG pathway enrichment analysis of untargeted metabolomic data from the serum of mice fed *C. butyricum*, we found that the intervention significantly reshaped the host's serum metabolic profile, with substantial alterations in multiple metabolic pathways (Figure S5c,d). Amino acid metabolism pathways (such as alanine, aspartate, and glutamate metabolism; arginine biosynthesis; glycine, serine, and threonine metabolism; histidine metabolism; and D‐amino acid metabolism), pyrimidine metabolism, mineral absorption, and a broad range of metabolic pathways all exhibited high enrichment scores and statistical significance. Notably, the GABAergic synapse pathway, which is closely associated with nervous system function, was also significantly enriched. This suggests that the *C. butyricum* intervention may have impacted the metabolic cycle of gamma‐aminobutyric acid (GABA), a major inhibitory neurotransmitter in the central nervous system. Concurrently, perturbations in a series of amino acid metabolic networks—particularly pathways involving excitatory neurotransmitters such as glutamate, aspartate, and glycine or their precursors—indicate a potential disruption in the balance of the neurotransmitter precursor pool. These systemic metabolic disturbances, especially the effects on GABAergic inhibitory signaling and glutamate‐related excitatory neurotransmitter metabolic networks, collectively point to a possible disturbance in the neural excitation‐inhibition balance. This provides potential mechanistic clues for explaining the observed phenotype of “declined cognitive function in mice” in the experiment: namely, that dysregulation of the aforementioned pathways may ultimately impair learning and memory by affecting synaptic transmission and neuronal excitability. It also suggests that gut microbiota may modulate the neurochemical environment of the gut‐brain axis via metabolites.

Notably, while our primary mechanistic focus was on *B. licheniformis*, the metabolomic data revealed that *C. butyricum* also induced a pronounced pro‐inflammatory profile and disrupted the neuroprotective balance, closely mirroring the effects observed with *B. licheniformis* (as summarized above). Both strains elicited a systemic metabolic shift characterized by the downregulation of neuroprotective and energy‐supportive metabolites (e.g., luteolin‐7‐glucoside, adenosine, 3‐hydroxybutyric acid) and the upregulation of potentially neurotoxic microbial‐derived metabolites (e.g., p‐cresol sulfate, phenyl sulfate). This convergence suggests that certain probiotic strains—despite taxonomic differences—may share common pathways in adversely modulating the gut–brain axis under chronic supplementation. The similar metabolic disruption pattern provides crucial clues for elucidating the potential metabolic mechanisms by which specific probiotics impact cognition, and highlights the need for strain‐specific, long‐term safety evaluations rather than generalizations about probiotic benefits.

To further investigate the systemic metabolic changes and regulatory relationships in mice following probiotics intervention, we constructed a metabolic regulatory interaction network based on the KEGG database using the screened differential metabolites. This network integrates multi‐layered information, including metabolic pathways, molecular modules, enzymes, chemical reactions, and metabolites, with the aim of revealing how specific metabolic perturbations propagate at the pathway level and the interactions between different pathways. The network analysis results are shown in Figure 5h and Figure S5e. In the figure, nodes of different categories are distinguished by color: red nodes represent affected metabolic pathways, yellow nodes represent regulatory enzymes associated with substance metabolism, green dots represent background metabolites for each pathway, purple nodes represent molecular functional modules, blue nodes indicate specific chemical reactions, and the key differential metabolites are highlighted as green squares. This regulatory network exhibits a high degree of complexity and connectivity. The differential metabolites (green squares) are widely distributed throughout the network and are directly connected via dense links to multiple metabolic pathways (red nodes) and specific biochemical reactions (blue nodes, such as R00677, R01361, R01317). This indicates that interventions with *B. licheniformis* and *C. butyricum* do not merely affect isolated metabolite levels but rather trigger extensive network‐wide perturbations involving multiple metabolic pathways. Differential metabolites often act as hubs connecting different pathway nodes, suggesting that these substances may be key junctions in the propagation of metabolic disturbances. Simultaneously, some regulatory enzymes (yellow nodes) are closely linked to differential metabolites and reaction nodes, implying that the activity of potential key enzymes may have been altered.

### 2.4 scRNA‐seq Reveals That Probiotic Treatment Remodels Hippocampal Cellular Composition

To elucidate the cellular heterogeneity and transcriptional landscape underlying the gut‐brain axis, we performed single‐cell RNA sequencing (scRNA‐seq) on 9 brain tissues from hippocampus of control vs. probiotic treatment mice using the 10x Genomics platform. After rigorous quality control to remove low‐quality cells and potential doublets, we obtained a high‐quality transcriptome dataset comprising 32,587 single cells from three biological replicates, with 13,069 cells in the *B. licheniformis* administrated mice, 11,156 cells in *C. butyricum* administrated mice, and 8,362 cells in the control group (Tables S6–S9). The sequencing data exhibited excellent quality, with a high median mapping rate to the mouse genome (approximately 96.5%) and Q30 (a Phred quality score threshold of 30) bases above 97%, ensuring the reliability of downstream analyses (Table S10 and Figure S6).

After aggregated and normalized scRNA‐seq data, t‐distributed stochastic neighbor embedding (t‐SNE) analysis using Seurat R Package was performed for cell‐type identification. Unsupervised clustering of the cells using Seurat revealed 16 distinct cell clusters for control group, 18 distinct cell clusters for *B. licheniformis* administrated group, and 16 distinct cell clusters for *C. butyricum* administered group (Figure S7).

We next annotated each cell type based on the expression level of canonical cell‐type‐specific markers (Table S11, Figures S8–S10). These clusters were annotated into major brain cell types based on the expression of well‐established marker genes recorded in the literature (Figure 6a–c, Figure S8 and Table S11). We identified ten major cell populations, including oligodendrocytes (OLGs), microglia (MG), endothelial cells (ECs), choroid plexus cells (CPCs), astrocyte (AC), pericytes, excitatory neuron (NC), B cells (BCs), T cells, and macrophages based on identified marker genes (Figure 6d).

**FIGURE 6:**
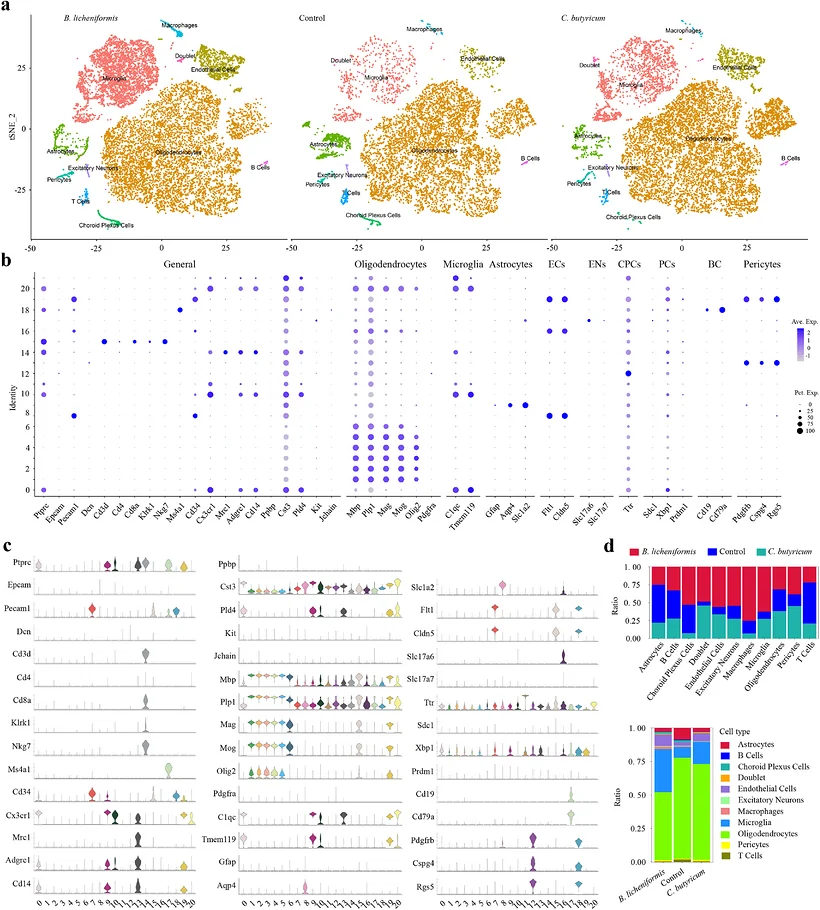
A single‐cell transcriptome atlas of hippocampus samples from probiotic‐administered mice. (a) t‐SNE plot visualizing cell clustering in hippocampal samples from the Control, *B. licheniformis*, and *C. butyricum* groups. Major cell populations are annotated, including microglia, macrophages, oligodendrocytes, astrocytes, endothelial cells, excitatory neurons, B cells, T cells, pericytes, and choroid plexus cells. (b) Dot plot displaying the average expression levels of key marker genes across different hippocampal cell types. The horizontal axis lists representative genes, and the vertical axis indicates cell identities (including General, Oligodendrocytes, Microglia, Astrocytes, ECs, ENs, CPCs, PCs, BC, and Pericytes). Dot size represents the percentage of cells expressing the corresponding gene within each cell type, while color intensity reflects the average expression level. Abbreviations: Pct. Exp., percentage of cells expressing the gene; Ave. Exp., average gene expression; ECs, endothelial cells; ENs, excitatory neurons; CPCs, choroid plexus cells; PCs, pericytes; BC, B cells. (c) Violin plots depicting the expression distribution of established cell‐type marker genes across the Control, *B. licheniformis*, and *C. butyricum* groups, which were used to determine cluster identities. The width of each violin corresponds to the density of gene expression, enabling visualization of both the central tendency and the spread of expression levels within each cell cluster. (d) Stacked bar graphs showing the proportional changes in major cell populations following probiotic administration (*B. licheniformis* and *C. butyricum*) compared to the Control group. The upper panel displays the relative ratio of specific cell types across the three groups, whereas the lower panel illustrates the overall cellular composition within each group. Cell populations include astrocytes, B cells, choroid plexus cells, doublets, endothelial cells, excitatory neurons, macrophages, microglia, oligodendrocytes, pericytes, and T cells, with distinct colors representing each cell type. Hippocampal tissues from 3 mice per group were pooled to construct one single‑cell RNA‑sequencing library for each experimental group.

Comparative analysis revealed significant differences in cell type abundance between the control and treatment groups, suggesting distinct functional alterations in the hippocampal microenvironment induced by the two bacterial treatments. The proportional distribution of ten major hippocampal cell types is summarized in Table S12 and S13. For Microglia, its proportion in the *B. licheniformis* group (32.18%) is approximately 4 times that in the Control group (8.03%), while the *C. butyricum* group (16.46%) shows a roughly twofold increase compared to the Control. Regarding Oligodendrocytes, the percentage in the *B. licheniformis* group (50.67%) is about 0.67 times that of the Control group (75.3%), and the *C. butyricum* group (71.36%) is nearly 0.95 times the Control level. In Endothelial Cells, the *B. licheniformis* group (8.47%) has a proportion about 3.44 times higher than the Control (2.46%), and the *C. butyricum* group (5.93%) is approximately 2.41 times that of the Control. As for Astrocytes, both the *B. licheniformis* (2.65%) and *C. butyricum* (2.7%) groups exhibit a proportion around 0.31 times that of the Control group (8.65%). These findings further indicate that long‐term probiotic consumption causes notable alterations in the microenvironment of the mice hippocampus.

### 2.5 Differential Gene Expression Analysis in Hippocampal After *B. licheniformis* Intervention

Compared to the control group, treatment with *B. licheniformis* yielded 3446 differentially expressed genes (DEGs) with statistically significant differential expression (adjusted *P*‐value < 0.05). Of these genes, the vast majority exhibited differences that were extremely significant (adjusted *P*‐value < 0.01), evidencing widespread changes at the transcriptome level (Figure 7a and Table S14). Among the genes with significant changes in expression levels, the number of upregulated genes (283, accounting for 8.2%) was notably higher than that of downregulated genes (113, accounting for 3.3%). Specifically, the *Hbb‐bs* gene showed the most dramatic upregulation (avg log_2_FC = 9.29) and was the most significantly differentially expressed gene (Table S14). Other significantly upregulated genes included *Hspa1a*/*b*, *Cd83*, I*er2*/*3*, *Fcrls*, *Fkbp5*, and *Nfkbiz* (Table S14). *Hbb‐bs* is a subunit of mouse hemoglobin, primarily responsible for oxygen transport. Its marked overexpression is often considered a marker of oxidative stress, tissue damage, or metabolic reprogramming. *Hspa1a*/*b* are members of the heat shock protein 70 family and belong to the classic cell stress‐protective proteins. Their high expression indicates that probiotic treatment triggers a significant cellular stress response, which may be a protective mechanism initiated by cells in response to environmental changes or internal inflammatory states. *Cd83* is an important activation marker for immune cells, particularly dendritic cells and B cells. Its pronounced overexpression directly suggests immune system activation and may enhance antigen presentation and immune responses. *Ier3* and *Ier2* are early response genes that can be rapidly induced by various stimuli, such as stress or growth factors, and are involved in the regulation of cell survival and apoptosis. The high expression of these two genes reflects a rapid transcriptional response of cells to probiotic stimulation. *Fcrls* is mainly expressed on macrophages, participating in the clearance of immune complexes and regulating immune responses. Its overexpression further supports the notion that immune cell function, especially that of macrophages, is modulated. *Fkbp5* is involved in glucocorticoid receptor signaling regulation and is closely associated with stress responses, inflammation, and immune modulation. Its high expression suggests that the neuroendocrine‐immune axis may be influenced by probiotics, thereby mediating anti‐inflammatory or stress adaptation processes. *Nfkbiz* is a key target gene and regulator of the NF‐κB signaling pathway, encoding the IκB‐ζ protein, which is a core mediator of inflammatory responses. Its significant overexpression strongly indicates marked activation of the NF‐κB inflammatory signaling pathway, a common mechanism through which probiotics exert immunomodulatory effects. The abnormal overexpression of these genes suggests that chronic exposure to *B. licheniformis* not only initiates a series of stress‐protective mechanisms, such as Hsp, but also significantly activates innate immunity and inflammation‐related signaling pathways, such as those involving Cd83 and Nfkbiz.

**FIGURE 7:**
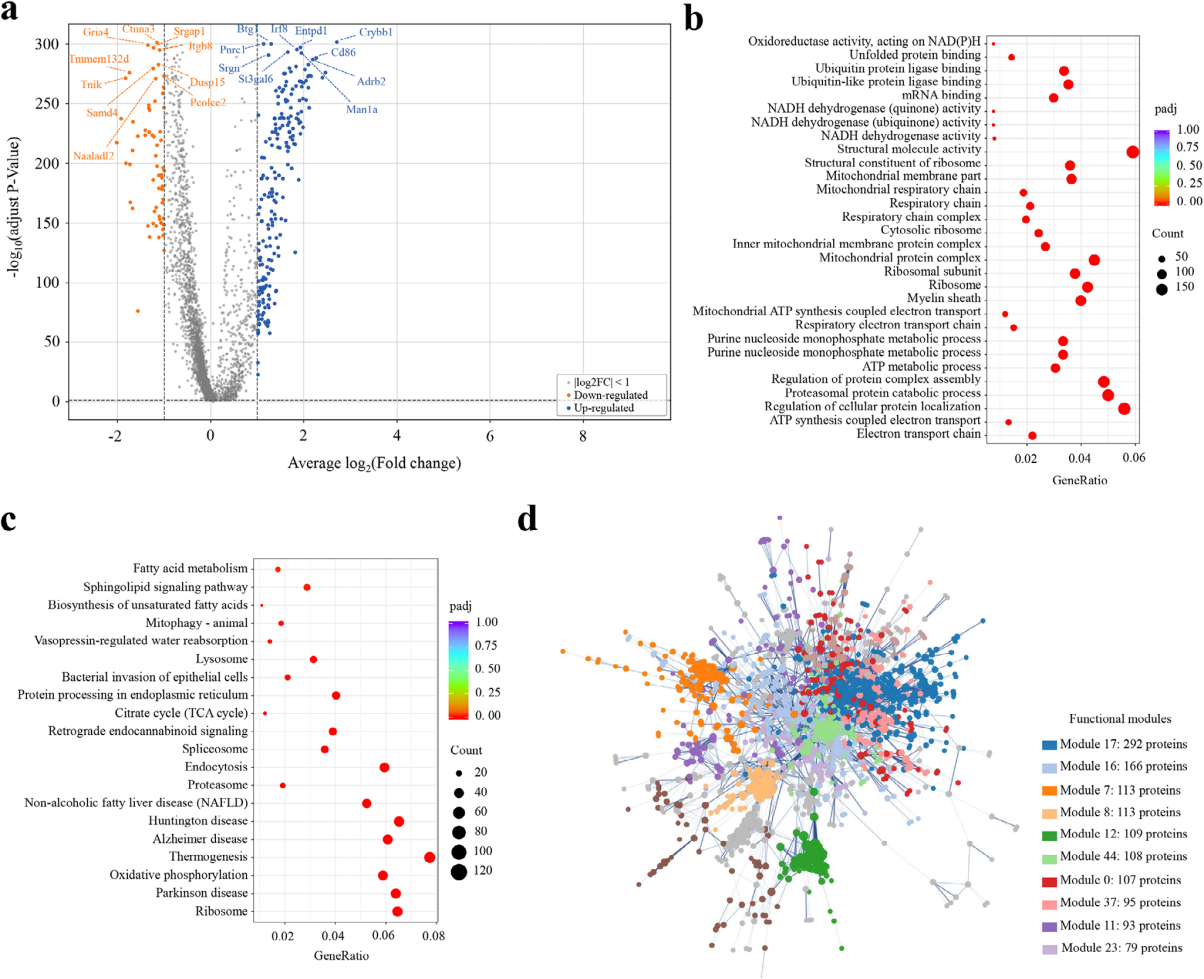
Functional and PPI analysis of hippocampal DEGs following *B. licheniformis* intervention. (a) Volcano plot displaying gene expression differences between groups, with thresholds set at an adjusted *P*‐value < 0.05. The x‐axis shows the average log_2_(fold change), while the y‐axis represents the ‐log_10_(adjusted *P*‐value). Data points are color‐coded: gray indicates non‐significant genes (|log_2_(fold change)| < 1), blue indicates up‐regulated genes, and orange indicates down‐regulated genes. Selected significantly differentially expressed genes (e.g., *Gria4*, *Cd86*, *Tmem132d*) are labeled. (b) GO functional clustering of DEGs between the *B. licheniformis* and control groups. (c) KEGG pathway enrichment analysis of these DEGs. (d) Protein‐protein interaction (PPI) network of DEGs. Nodes are color‐coded to represent distinct functional modules. Major modules include Module 17 (292 proteins; e.g., Ctnnb1, Kras, Hras) and Module 16 (166 proteins; e.g., Hsp90ab1, Hspa8, Dync1h1). Additional modules of interest are Module 7 (113 proteins; e.g., Snrpd3, Sf3b1, Prpf8) and Module 8 (113 proteins; e.g., Rps5, Rps3, Rps11). The network also highlights Module 12 (109 proteins; e.g., Ndufs3, Uqcrc2, Ndufa5), Module 44 (108 proteins; e.g., Ubb, Rps27a, Pten), Module 0 (107 proteins; e.g., Fos, Ep300, Kat2b), Module 37 (95 proteins; e.g., Smarca4, Actl6a, Cops5), Module 11 (93 proteins; e.g., Rab28, Hadh, Acat1), and Module 23 (79 proteins; e.g., Sec13, Chmp2a, Chmp4b).

In contrast, the transcription levels of genes such as *Hcn2*, *Grm7*, *Robo1*, *Nrxn1*, *Tenm2*, *Adgrb3*, *Npas3*, and *Tmem132d* were significantly downregulated (Figure 7a and Table S14). Among these, *Hcn2* encodes the hyperpolarization‐activated cyclic nucleotide‐gated channels, and *Grm7* encodes a metabotropic glutamate receptor, both of which jointly regulate neuronal excitability and synaptic transmission. This result suggests that probiotic treatment may indirectly affect the electrical activity of neurons or neural signaling processes. Robo1 and Nrxn1 are key molecules involved in axon guidance and synapse formation, playing crucial roles in nervous system development and neural circuit assembly. The downregulation of these genes in non‐developmental adult tissues may reflect altered neuroplasticity‐related processes. Tenm2 and Adgrb3 are involved in cell adhesion and synaptic organization, playing important roles in maintaining neural network structure. Together with the above genes, they suggest that pathways related to neural connectivity or intercellular interactions may be suppressed. Additionally, Npas3 and Tmem132d are associated with brain development, cognitive function, and the risk of psychiatric disorders. Collectively, this cluster of downregulated genes strongly points toward neural‐related functions, further indicating that probiotics have a significant impact on neural gene expression.

Gene Ontology (GO) functional enrichment analysis was performed to characterize the differentially expressed genes (DEGs) between the *B. licheniformis* and control groups. The overall DEGs were significantly enriched in multiple biological processes, cellular components, and molecular functions (Figure 7b). Specifically, the up‐regulated DEGs were primarily associated with ribosomal structure and translation, including “structural constituent of ribosome”, “ribosome”, “cytosolic ribosome”, and “cytoplasmic translation”, as well as protein complex assembly and actin polymerization (Figure S11a). Conversely, the down‐regulated DEGs were mainly involved in mitochondrial respiration and energy metabolism, such as “electron transport chain”, “mitochondrial respiratory chain”, “ATP synthesis coupled electron transport”, and “NADH dehydrogenase activity” (Figure S11b).

KEGG pathway enrichment analysis was conducted to further investigate the biological pathways associated with the DEGs between the *B. licheniformis* and control groups. The overall DEGs were significantly enriched in several key pathways, including “Ribosome”, “Oxidative phosphorylation”, “Thermogenesis”, “Alzheimer disease”, “Huntington disease”, “Non‐alcoholic fatty liver disease (NAFLD)”, and “Fatty acid metabolism” (Figure 7c). Specifically, the up‐regulated DEGs were predominantly enriched in the “Ribosome” pathway, which exhibited the highest GeneRatio (approximately 0.15) and Count. Other notable enriched pathways for up‐regulated genes included “Antigen processing and presentation”, “Protein processing in endoplasmic reticulum”, “Autophagy‐animal”, “Phagosome”, and various infection‐related pathways such as “Viral carcinogenesis” and “Salmonella infection” (Figure S12a). Conversely, the down‐regulated DEGs were mainly associated with “Oxidative phosphorylation”, “Thermogenesis”, “Alzheimer disease”, “Huntington disease”, “Parkinson disease”, and “NAFLD”. Additionally, pathways related to metabolic processes, such as “Fatty acid metabolism”, “Citrate cycle (TCA cycle)”, and “Carbon metabolism”, were also significantly enriched in the down‐regulated gene set (Figure S12b).

The high‐confidence Protein‐Protein Interaction (PPI) network constructed in this study exhibited typical scale‐free and modular characteristics (Figure 7d). A total of 1,797 proteins formed a highly integrated network with 7,980 interactions, dominated by the giant connected component accounting for 91.3% of all nodes. Hub proteins of the network were mainly ribosomal proteins and ubiquitin‐system proteins. Among them, Ubb possessed both high degree and high betweenness centrality, serving as a core node linking translation, degradation, and signaling pathways. Louvain community detection identified 74 functional modules, which were predominantly enriched in five major functional groups: gene expression, protein quality control, energy metabolism, signal‐transduction, and intracellular transport. Inter‐module interaction analysis revealed that the signal‐transduction module was the most widely connected functional unit within the network, forming extensive crosstalk with modules such as protein degradation, transcriptional regulation, and molecular chaperones.

### 2.6 Differential Gene Expression Analysis in Major Hippocampal Cell Types Following *B. licheniformis* Intervention

To further dissect the cellular heterogeneity of the transcriptomic response, we mapped the distribution of differentially expressed genes (DEGs) across 10 major hippocampal cell types, identifying a total of 4,958 DEGs (Figure 8a). Oligodendrocytes emerged as the most responsive population, harboring 3,087 DEGs, followed by astrocytes (2,524) and microglia (770). Moderate responses were observed in endothelial cells (110) and macrophages (94), with choroid plexus cells showing 37 DEGs. In contrast, B cells, pericytes, excitatory neurons, and T cells displayed relatively muted transcriptional shifts.

**FIGURE 8:**
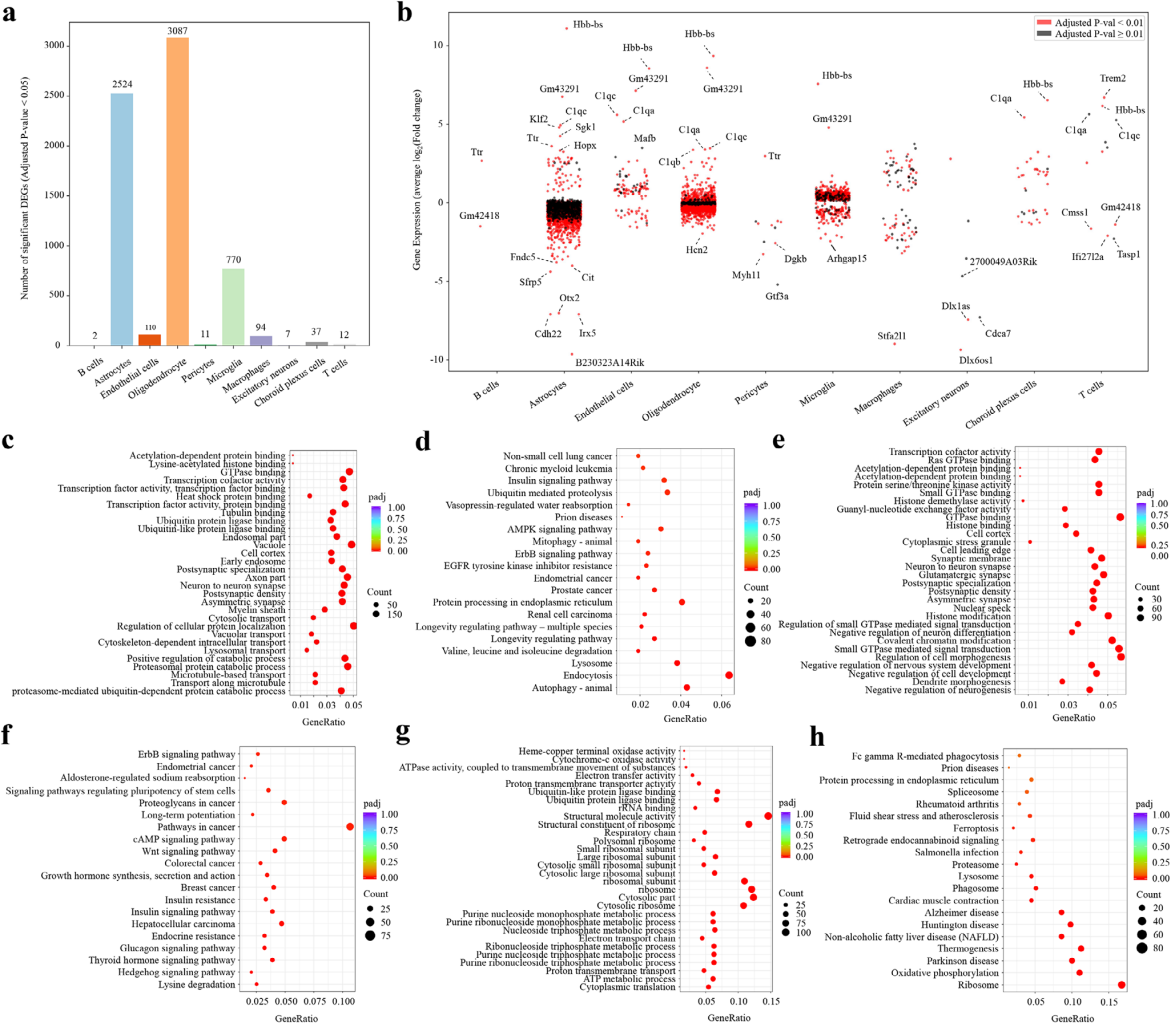
Cell‐type‐specific differential gene expression and functional enrichment in *B. licheniformis*‐intervened mice. (a) Bar chart showing the distribution of the number of significant DEGs (adjusted *P*‐value < 0.05) across 10 major cell types in *B. licheniformis*‐treated mice. The y‐axis indicates the number of significant DEGs. Oligodendrocytes exhibit the highest number of DEGs (3087), followed by astrocytes (2524) and microglia (770). (b) Band plots illustrating the distribution of DEGs (dots) across the 10 major cell types, with the y‐axis representing gene expression (average log_2_(fold change)). Significant DEGs (adjusted *P*‐value < 0.01 and |average log_2_(fold change)| > 0.25) are plotted in red, whereas black dots denote genes that did not meet these significance thresholds. Selected key genes (e.g., *Hbb‐bs*, *C1qa*, *Ttr*, *Trem2*) are labeled. (c) GO functional clustering of DEGs in oligodendrocytes. (d) Corresponding KEGG pathway enrichment analysis. (e) GO functional clustering of DEGs in astrocytes. (f) Corresponding KEGG pathway enrichment analysis. (g) GO functional clustering of DEGs in microglia. (h) Corresponding KEGG pathway enrichment analysis. For the bubble charts (c–h), the x‐axis represents the GeneRatio, the dot size indicates the number of enriched genes (Count), and the color gradient reflects the adjusted *P*‐value (padj).

Regarding expression directionality, microglia, endothelial cells, and macrophages were predominantly characterized by upregulation, whereas oligodendrocytes and astrocytes primarily exhibited downregulated transcripts (Figure 8b). Notably, *Ttr* and *Gm42418* were perturbed in nearly all cell types examined, suggesting their potential role as master molecular “switches” mediating the effects of *B. licheniformis*. The robust upregulation observed in immune‐related cell types (microglia and macrophages) implies an enhancement of neuroimmune surveillance. Concurrently, the prominent downregulation in oligodendrocytes may reflect the engagement of neuroprotective mechanisms. In summary, *B. licheniformis* exerts markedly heterogeneous effects across brain cell types, with the most pronounced impact on oligodendrocytes and astrocytes, potentially conferring neuroprotection via activation of shared immune and gene regulatory pathways.

To characterize the functional implications of these transcriptional changes, GO enrichment analysis was performed on oligodendrocyte DEGs. The overall gene set was significantly enriched across diverse biological processes, cellular components, and molecular functions (Figure 8c). Upregulated genes were strongly associated with proteostasis, including “protein folding,” “‘de novo’ posttranslational protein folding,” and “chaperone binding,” alongside mitochondrial respiration (“mitochondrial respiratory chain”) and lysosomal components (Figure S13a). Conversely, downregulated genes were enriched in cytoskeletal transport (“microtubule‐based transport,” “Golgi vesicle transport”) and synaptic architecture (“postsynaptic density,” “asymmetric synapse”), as well as GTPase binding (Figure S13b). KEGG pathway analysis further revealed significant enrichment in “Autophagy—animal,” “Endocytosis,” and “Protein processing in endoplasmic reticulum,” alongside disease pathways such as “Alzheimer disease” (Figure 8d). Upregulated pathways highlighted cellular turnover and signaling (“Wnt signaling pathway”), whereas downregulated pathways pointed to metabolic suppression (“Oxidative phosphorylation”) and disease associations (Figure S14a,b). Notably, “Protein processing in endoplasmic reticulum” (down‐regulated) exhibited both high enrichment ratios and a substantial gene count.

In astrocytes, GO analysis revealed a distinct functional landscape (Figure 8e). Upregulated genes were predominantly linked to glial development and myelination, including “oligodendrocyte differentiation,” “myelination,” and “axon ensheathment,” coupled with tissue repair responses (“response to wounding”) (Figure S15a). In contrast, downregulated genes were concentrated in synaptic transmission (“glutamatergic synapse”) and the negative regulation of neural development, alongside small GTPase signaling (Figure S15b). KEGG profiling indicated enrichment in cancer and signaling pathways (“Wnt signaling pathway,” “Insulin signaling pathway”) (Figure 8f). Upregulation was associated with oncogenic pathways (“Hepatocellular carcinoma”) and long‐term potentiation, while downregulation affected metabolic pathways (“Pyruvate metabolism”) and cytoskeletal regulation (“Regulation of actin cytoskeleton”) (Figure S16a,b). Core pathways such as “Pathways in cancer” and “Proteoglycans in cancer” showed high enrichment ratios.

Finally, in microglia, GO terms highlighted a polarization toward translation and metabolism (Figure 8g). Upregulated genes were enriched in ribosomal structure (“cytosolic ribosome”) and ATP metabolism, whereas downregulated genes involved GTPase signaling (“Ras GTPase binding”) and postsynaptic organization (Figure S17a,b). KEGG analysis corroborated these findings, showing enrichment in “Ribosome” and “Oxidative phosphorylation” (Figure 8h). Upregulated pathways included immune activation (“NF‐kappa B signaling pathway”), while downregulated pathways involved proteostasis (“Proteasome”) and neurodegenerative diseases (“Alzheimer disease”). The “Ribosome” pathway was a dominant feature across both overall and downregulated gene sets, indicating a significant shift in protein synthesis capacity.

## 3 Discussion

The widespread use of probiotics is predicated on their purported health benefits, particularly for gastrointestinal and cognitive function. However, the long‐term effects and potential risks of chronic probiotic supplementation, especially in the context of the gut‐brain axis, remain incompletely understood. This study presents compelling evidence that chronic oral administration of specific probiotics, notably *B. licheniformis*, can induce significant cognitive decline in mice. This impairment appears mechanistically associated with a triad of pathological events: profound gut microbiota dysbiosis, systemic metabolic perturbations, and hippocampal neuroinflammation accompanied by cellular remodeling. Our findings challenge the simplistic paradigm of probiotics as universally beneficial and underscore the importance of strain‐specific and context‐dependent evaluations.

Our initial behavioral and histopathological analyses revealed a surprising outcome: six‐month administration of *B. licheniformis*, *C. butyricum*, and *L. acidophilus* led to significant deficits in social ability and memory, alongside marked hippocampal neurodegeneration, including reduced neuronal soma size, dendritic complexity, and spine density. While *L. acidophilus* showed a milder effect on social memory, the structural damage was consistent across all treated groups. This indicates that chronic probiotic intervention, under our experimental conditions, exerted adverse effects on synaptic integrity and neuronal health, providing a structural basis for the observed cognitive dysfunction.

We observed that mice administered *B. cereus* and *B. longum* subsp. *longum* exhibited rapid and progressive body weight loss preceding mortality, suggesting potential strain‐specific toxicity under our experimental conditions. While the precise mechanisms underlying these adverse outcomes warrant further investigation, these findings underscore the critical importance of strain‐specific characterization in probiotic research. It is important to note that the vast diversity of probiotic strains—coupled with the fact that many commercial strains lack fully transparent genomic and phenotypic characterization—precludes exhaustive, systematic evaluation of all candidates within a single study. Consequently, our experimental design prioritized a comprehensive, mechanistic assessment of *B. licheniformis*, given its pronounced effects on cognition and hippocampal structure. The inclusion of other probiotic strains, including *B. cereus* and *B. longum* subsp. *longum*, served primarily as comparative benchmarks to contextualize the effects of *B. licheniformis*. Therefore, the severe adverse events observed with these specific strains should not be extrapolated to all members of their respective species or to probiotics in general, but rather highlight the inherent variability and potential risks associated with unsupervised, long‐term administration of inadequately characterized strains.

A central finding of this study is that the observed central nervous system (CNS) effects are likely mediated through the gut‐brain axis, initiated by a profound disruption of gut microbial ecology [40]. Metagenomic sequencing revealed that each probiotic strain induced a unique and significant shift in gut microbiota composition and functional profile. Notably, treatment with *C. butyricum*, *B. licheniformis*, and *L. acidophilus* all resulted in a marked reduction in the abundance of endogenous *L. acidophilus* within the murine intestine. Given established literature linking the abundance of this specific bacterium to cognitive function, this depletion may represent a critical mechanism underlying the observed neurological alterations [16, 41, 42]. Furthermore, treatment with *B. licheniformis* was associated with a distinct shift in the microbial community structure, characterized by an enrichment of nucleotide metabolism pathways. In contrast, exogenous administration of *L. acidophilus* and *C. butyricum* drove community composition toward functions related to amino acid metabolism and transport. Over‐activation of nucleotide metabolism (e.g., purine metabolism) can lead to excessive production of ATP and its derivatives (adenosine). While adenosine is neuroprotective in acute settings, chronic elevation can disrupt the purinergic signaling system, potentially leading to neuroinflammation and excitotoxicity, which are hallmarks of cognitive impairment [43, 44]. Nucleotides are key signaling molecules. Alterations in microbial nucleotide metabolism can influence systemic inflammation [45]. High levels of circulating nucleotides or their metabolites (like uric acid) have been correlated with oxidative stress in the brain, contributing to deficits in memory and learning [46, 47, 48]. Amino acids are direct precursors to neurotransmitters. A shift toward amino acid metabolism, particularly tryptophan metabolism via the kynurenine pathway, is a double‐edged sword. While it can produce neuroactive metabolites (e.g., kynurenic acid), excessive flux through this pathway (often induced by inflammation) can deplete tryptophan for serotonin synthesis and produce neurotoxic metabolites (e.g., quinolinic acid), leading to cognitive deficits [49, 50, 51]. This indicates that probiotics are not passive passengers but active “eco‐system engineers” that can remodel the gut environment in potentially undesirable directions. The dysbiosis induced by *B. licheniformis* was not merely compositional but also functional, potentially altering the gut's metabolic output.

Consistent with this, untargeted serum metabolomics confirmed that *B. licheniformis* and *C. butyricum* treatments induced widespread systemic metabolic reprogramming. Key altered pathways included central carbon metabolism, amino acid and nucleotide biosynthesis, and ABC transporter activity. Notably, metabolites related to lipid metabolism and microbial fermentation (e.g., specific carnitines [52], butyrate [53]) were significantly changed. This systemic metabolic disorder represents a critical link, as these circulating metabolites can cross the blood‐brain barrier or signal via humoral pathways to influence brain function [54]. The enrichment of pathways like “central carbon metabolism in cancer” [55, 56] and “protein digestion and absorption” [57] suggests a state of metabolic stress and altered nutrient sensing in the host, which can have profound implications for brain energy homeostasis and neuroinflammation.

The metabolic perturbations identified in this study—spanning lipid, amino acid, and nucleotide metabolism—provide critical mechanistic insights into how peripheral metabolic dysregulation propagates to hippocampal dysfunction and neuroinflammation. Regarding nucleotide metabolism, the *B. licheniformis*–induced enrichment of nucleotide metabolism pathways (e.g., pyrimidine metabolism, base excision repair) at the metagenomic level was directly paralleled by elevated serum purine metabolites, particularly adenosine 3′‐monophosphate. While acute adenosine signaling is neuroprotective, chronic elevation of purine metabolites can disrupt purinergic receptor signaling (P1 and P2 receptors), promoting neuroinflammation and excitotoxicity—hallmarks of cognitive impairment. This is consistent with our scRNA‐seq data showing activation of NF‐κB and TNF signaling pathways in hippocampal microglia, as well as upregulation of oxidative stress markers (*Hspa1a*/*b*, *Hbb‐bs*). Regarding amino acid metabolism, significant alterations in pathways such as alanine, aspartate, and glutamate metabolism, glycine/serine/threonine metabolism, and arginine biosynthesis have profound implications for neurotransmitter homeostasis. Glutamate, the major excitatory neurotransmitter, relies on balanced precursor availability; disruption of its metabolic cycle can impair synaptic vesicle function and NMDA receptor activity. Furthermore, the observed alterations in D‐amino acid metabolism and, in the *C. butyricum* group, GABAergic synapse pathways suggest a potential imbalance in the excitation–inhibition equilibrium essential for normal cognitive processing. The downregulation of synaptic genes (*Hcn2*, *Grm7*, *Robo1*, *Nrxn1*) in our scRNA‐seq data provides a direct transcriptomic correlate to these amino acid–mediated synaptic disturbances. Regarding lipid metabolism, untargeted metabolomics revealed significant changes in metabolites related to mitochondrial function (e.g., tetradecenoyl carnitine, 3‐hydroxybutyric acid) and neuroprotection (e.g., naringenin, luteolin‐7‐glucoside). The downregulation of 3‐hydroxybutyric acid—a ketone body that serves as an alternative energy substrate for the brain—suggests restricted cerebral energy supply, which may exacerbate neuronal vulnerability. Concurrently, the downregulation of fatty acid metabolism pathways in oligodendrocytes (scRNA‐seq) indicates impaired myelin lipid synthesis, potentially compromising axonal signal transmission efficiency. Collectively, these interconnected metabolic disturbances—nucleotide‐driven neuroinflammation, amino acid–mediated neurotransmitter imbalance, and lipid‐related energy deficit—converge to create a hippocampal microenvironment characterized by oxidative stress, synaptic dysfunction, and glial reactivity, ultimately culminating in the observed cognitive decline.

The consequences of this gut‐derived dysbiosis and metabolic disturbance were clearly evident at the level of the hippocampus. Single‐cell RNA sequencing revealed a dramatic remodeling of the hippocampal cellular landscape following probiotic treatment. The most striking change was a massive expansion of the microglial population (a ∼4‐fold increase in the *B. licheniformis* group), coupled with a reduction in oligodendrocytes and astrocytes. This shift indicates a robust neuroinflammatory response and a potential loss of crucial supportive functions for neurons [58, 59, 60, 61]. Differential gene expression analysis across cell types provided deep mechanistic insights. In microglia, upregulated genes pointed toward an activation state focused on protein clearance and response to neurodegenerative cues (e.g., enrichment in Alzheimer's and prion disease pathways), alongside signs of cellular stress. This suggests microglia are attempting to cope with a pathological brain environment, potentially triggered by peripheral signals [62, 63].

Even more telling were the changes in oligodendrocytes and astrocytes. Oligodendrocytes showed a widespread downregulation of genes essential for their supportive functions, including fatty acid metabolism (critical for myelination) and pathways for structural connectivity (adherens junction, axon guidance). Concurrently, they upregulated pathways associated with proteotoxic stress, endoplasmic reticulum stress, and surprisingly, antigen presentation. This paints a picture of oligodendrocytes transitioning from a myelinating, supportive state to a stressed, dysfunctional, and potentially immunogenic one. Similarly, astrocytes exhibited a downregulation of homeostatic and synaptic support genes, while upregulating a suite of immune‐inflammatory and stress‐response pathways, hallmarking a transition to a pro‐inflammatory, reactive state. The pronounced downregulation of neuronal genes involved in synaptic transmission (e.g., *Grm7*, *Nrxn1*) and ion channel function (*Hcn2*) provides a direct molecular correlate to the observed synaptic structural deficits and cognitive impairment [64, 65, 66].

Collectively, our multi‐omics data support a coherent pathological cascade. Chronic *B. licheniformis* intervention first induces specific gut microbiota dysbiosis, altering the functional potential of the microbial community. This dysbiosis leads to systemic metabolic perturbations, disrupting host energy, amino acid, and lipid metabolism. These aberrant microbial metabolites and systemic signals, via circulatory and neural pathways, reach the hippocampus. There, they trigger a pronounced neuroinflammatory response, characterized by microglial proliferation and the reactive transformation of astrocytes and oligodendrocytes. This inflamed and dysfunctional brain microenvironment, featuring disrupted synaptic gene expression, oxidative stress (evidenced by *Hsp* [67] and *Hbb‐bs* [68] upregulation), and impaired supportive glial function, ultimately culminates in neuronal structural damage and cognitive decline [59, 69].

To substantiate the multi‐omics findings, we leveraged our existing experimental data across behavioral, histological, and multi‐omics modalities. However, due to the pooled‐sample design for metagenomic and scRNA‐seq data, these integrations are limited to group‐level comparisons and should be interpreted as convergent observational evidence rather than statistically correlated relationships. First, the cognitive impairment quantified by the three‐chamber test (Figure 2) is consistent with the downregulation of key synaptic genes (e.g., *Hcn2*, *Grm7*, *Robol*, *Nrxn1*) in the scRNA‐seq dataset, **s**uggesting a potential transcriptomic correlate to synaptic protein loss and structural deficits. Second, the hippocampal structural abnormalities revealed by Golgi staining—including reduced neuronal density, soma size, dendritic length, and spine density—provide convergent histological support for the scRNA‐seq cellular landscape. The ∼4‐fold expansion of the microglial population (32.18% in *B. licheniformis* vs. 8.03% in control; Table S12) parallels the expected Iba1+ microglial proliferation, while the transcriptional shift of astrocytes toward a pro‐inflammatory reactive state (upregulation of immune and stress‐response pathways, Figure S16) parallels GFAP+ astrogliosis. Similarly, the scRNA‐seq observed decrease in oligodendrocyte abundance aligns with the impaired homeostatic transcriptional profile and the Golgi evidence of reduced neuronal support, consistent with Olig2+ cell decline. Third, the metagenomic prediction of enriched nucleotide metabolism in the *B. licheniformis* group is consistent with the serum metabolomic detection of elevated purine‐related metabolites (e.g., adenosine 3'‐monophosphate), suggesting a potential link between microbial functional shifts and host metabolic perturbations. Fourth, the serum metabolomic alterations in neuroprotective and neurotoxic metabolites (e.g., down‐regulation of naringenin and 3‐hydroxybutyric acid; up‐regulation of 2‐methylhippuric acid, p‐cresol glucuronide, and p‐cresol sulfate) are consistent with the scRNA‐seq observations of neuroinflammatory pathway activation, including NF‐κB signaling (evidenced by upregulation of *Nfkbiz*, *Cd83*, *Fcrls*, and *Fkbp5*) and oxidative stress markers (*Hspa1a*/*b*, *Hbb‐bs*). While pro‐inflammatory cytokines (IL‐1β, TNF‐α, IL‐6) were not quantified at the protein level, the activation of NF‐κB and TNF signaling pathways in microglia (Figure S18) and the systemic accumulation of bacterial uremic toxins strongly suggest a pro‐inflammatory milieu. Finally, as detailed in the Limitations section, formal statistical correlation or network analyses integrating all multi‐omics datasets with neurobehavioral outcomes could not be performed due to the pooled‐sample design. Nevertheless, the convergent lines of evidence from independent assays—spanning behavioral, histological, metagenomic, metabolomic, and transcriptomic modalities—collectively support the hypothesis of a gut‐brain axis cascade. These convergent lines of evidence provide suggestive support for the identified inflammatory and metabolic pathways, even in the absence of targeted protein‐level validation for markers such as NF‐κB, IBA1, GFAP, and synaptic proteins.

While our integrated multi‐omics approach provides a comprehensive, systems‐level view of gut‐brain axis alterations induced by chronic *B. licheniformis* exposure, several limitations warrant consideration. First, this research was conducted in a specific pathogen‐free (SPF) mouse model under conditions of long‐term, high‐dose administration. Consequently, the generalizability of these findings to humans or to animals with distinct baseline microbiomes, underlying pathologies, or different dosing regimens remains to be established. Second, although single‐cell RNA sequencing offers high‐resolution insights into cellular states and pathway activation, transcriptomic changes do not always correlate directly with protein expression levels. Targeted protein validation—via Western blotting (WB), enzyme‐linked immunosorbent assay (ELISA), or immunohistochemistry/immunofluorescence for key neuroinflammatory markers (e.g., NF‐κB, IBA1, GFAP, and cytokines)—will be essential to consolidate the translational relevance of our findings. Similarly, while our untargeted metabolomics approach identified widespread metabolic perturbations, targeted metabolomics with authentic standards (e.g., multiple reaction monitoring) for absolute quantification of key metabolites—such as adenosine 3′‐monophosphate, p‐cresol sulfate, 2‐methylhippuric acid, and 3‐hydroxybutyric acid—will be required to confirm the present findings and establish precise concentration–response relationships with cognitive outcomes. Third, shotgun metagenomic and hippocampal single‐cell RNA‐sequencing data were generated from group‐pooled biological samples, producing only one aggregated sequencing profile for each experimental condition. Rigorous correlation or multi‐omics network analysis demands paired multi‐omics and behavioral measurements from independent biological replicates (typically 3–5 replicates per group) at the individual‐animal level. In addition, the single pooled metagenomic sample per group prevents robust assessment of within‐group gut‐microbiota variability and precludes replicate‐based differential‐abundance tools including LEfSe, limiting statistical interpretation of alpha‐ and beta‐diversity metrics. Given that we lack individual‐mouse‐resolved microbiome and hippocampal transcriptome datasets, formal individual‐animal‐level correlative or integrative multi‐omics network analyses cannot be performed, and we avoid presenting potentially inflated or misleading correlative claims based on inadequate replication. Future studies adopting non‐pooled, individual‐mouse multi‐omics profiling will be a high‐priority direction to construct quantitative correlative networks and further validate the gut‐brain‐axis‐associated molecular interrelationships observed in this work. Fourth, while our in vivo model captures the systemic complexity of host‐microbe interactions, it cannot fully dissect the direct cellular mechanisms by which *B. licheniformis* or its metabolites influence human brain‐resident immune cells. Complementary in vitro investigations, such as exposing human HMC3 (human microglial clone 3) microglial cells to bacterial conditioned media, are required to elucidate these direct neuroinflammatory signaling cascades. Finally, while we establish strong correlative links between microbial shifts and hippocampal phenotypes, definitive causal relationships require validation through targeted interventions, such as fecal microbiota transplantation or metabolite supplementation studies. Future research addressing these gaps will be critical to confirm the proteomic consequences of transcriptional shifts and to validate both the direct effects on human microglial function and the causal microbial drivers of the observed cognitive deficits.

In conclusion, this work provides a critical counterpoint to the prevailing narrative of probiotic benefits. We demonstrate that chronic supplementation with certain probiotic strains, particularly *B. licheniformis*, can have deleterious effects on cognition by orchestrating a detrimental cascade from the gut to the brain. This effect is mediated through driving gut microbiota dysbiosis, instigating host metabolic disorder, and ultimately fueling hippocampal neuroinflammation and cellular dysfunction. These findings emphasize that probiotics are potent biological agents with complex, strain‐specific effects. Their therapeutic application, especially for long‐term cognitive support, must be guided by rigorous, individualized safety and efficacy assessments rather than a presumption of universal benefit. Future research should focus on identifying the specific microbial metabolites and signaling molecules responsible for these effects and defining the host factors that determine susceptibility to such adverse outcomes.

## 4 Methods

### 4.1 Animal Model and Experimental Design

A schematic overview of the experimental design and study workflow is provided in Figure 1. A total of 90 adult male C57BL/6J mice (8 weeks old) were acquired from Hangzhou Wenjing Biotechnology Co., Ltd. (Hangzhou, China) and maintained under standardized housing conditions (12‐h light/dark cycle, ambient temperature 22°C ± 1°C, ad libitum access to food and water). Following a one‐week acclimation period, mice were randomized into six groups (*n* = 15/group): one vehicle control group and five probiotic intervention groups. The control group received daily oral gavage of sterile phosphate‐buffered saline (PBS), while the five experimental groups received daily oral gavage with distinct commercial probiotic formulations for six consecutive months. The specific probiotic strains—*B. licheniformis* (ATCC14580), *C. butyricum* (ATCC19398), *B. cereus* (ATCC14579), *L. acidophilus* (ATCC4356), and *B. longum* subsp. *longum* (ATCC55813)—and their commercial sources are summarized in Table S15. All products were purchased from Shanghai Microbial Preservation Co., Ltd. (Shanghai, China), prepared according to the manufacturers' instructions, and delivered at a standardized daily dose of 1 × 10^7^ colony‐forming units (CFU) per mouse. Cognitive performance was assessed during the final week using the three‐chamber social approach test. Upon completion of the intervention, fecal pellets, serum, and hippocampal tissues were collected for multi‐omics analyses, including shotgun metagenomic sequencing of gut microbiota (fecal DNA; Illumina HiSeq), untargeted metabolomics of serum (UHPLC‐Orbitrap LC‐MS/MS), single‐cell RNA sequencing of hippocampal tissue (10x Genomics), and histomorphological assessment (Golgi staining). Integrated analysis across all omics layers elucidated the mechanistic cascade linking gut microbiota dysbiosis to hippocampal neuroinflammation and cognitive decline. The entire experimental procedure was performed in strict compliance with the guidelines approved by the Medical Ethics Committee of Huzhou Normal University (Approval No. 20240318).

### 4.2 Behavioral Testing: Three‐Chamber Social Approach Test

Cognitive and social behavioral assessments were conducted during the final week of the six‐month feeding regimen. The evaluation was performed using a modified version of the three‐chamber social interaction test, as previously described in the literature [70]. The testing protocol consisted of three sequential phases: habituation, sociability test, and social novelty test. First, the experimental mouse was allowed to freely explore all three interconnected, empty chambers of the apparatus for a habituation period. For the sociability phase, an unfamiliar, age‐matched stimulus mouse (stranger 1) was enclosed within a small wire cage placed in one side chamber, while an identical but empty wire cage was placed in the opposite chamber. The test mouse was then allowed to explore, and its interaction with the social target (stranger 1) versus the non‐social object (empty cage) was measured. Subsequently, for the social novelty phase, a second unfamiliar mouse (stranger 2) was introduced into the previously empty wire cage. The test mouse's interaction with the now‐familiar mouse (stranger 1) versus the novel mouse (stranger 2) was assessed to evaluate social recognition memory. All sessions were video‐recorded. Parameters including the time spent in each chamber, the duration of direct sniffing interaction with each wire cage, and the number of entries into each chamber were automatically tracked and quantified using a video tracking system. Based on this data, preference indices were calculated for both the sociability and social novelty phases to objectively quantify social approach behavior and social recognition memory, respectively.

### 4.3 Sample Collection

Upon completion of the six‐month experimental period, a comprehensive sample collection and tissue processing procedure was conducted. Fresh fecal pellets were collected directly from each mouse, immediately snap‐frozen in liquid nitrogen, and stored at ‐80°C for subsequent metagenomic analysis of the gut microbiota. Following this, mice were deeply anesthetized. Blood samples were then obtained via cardiac puncture. The collected blood was centrifuged to separate plasma, which was aliquoted and stored at ‐80°C for global, untargeted metabolomic profiling.

Subsequently, for brain tissue preservation, the mice were transcardially perfused. Perfusion was initiated with ice‐cold phosphate‐buffered saline (PBS) to flush out blood components, followed by fixation with ice‐cold 4% paraformaldehyde (PFA) in PBS. The brains were carefully dissected out and then post‐fixed in 4% PFA at 4°C for 24 h. After fixation, brains were cryoprotected by immersion in a 30% sucrose solution in PBS at 4°C until they sank. The cryoprotected brains were then sectioned coronally at a specified thickness using a freezing microtome or cryostat for histological and immunofluorescence staining.

For the hippocampus specifically, two processing pathways were employed. For morphological and protein‐level analysis, hippocampal regions were either processed for paraffin embedding or optimal cutting temperature (OCT) compound embedding for cryosectioning, followed by various microscopic examinations. For transcriptomic analyses, fresh hippocampal tissue was rapidly dissected on ice from a separate cohort of non‐perfused mice. This tissue was either flash‐frozen for bulk RNA analysis or immediately subjected to tissue dissociation to create a single‐cell suspension, which was then processed for droplet‐based single‐cell RNA sequencing (scRNA‐seq) to profile cell‐type‐specific transcriptional changes.

### 4.4 Histological Staining

Coronal brain sections, 30 µm in thickness, encompassing the hippocampal region were prepared for histological analysis. To analyze dendritic arborization and spine morphology, a separate set of brain sections containing the hippocampus was processed using the FD Rapid GolgiStain Kit (FD NeuroTechnologies), strictly adhering to the manufacturer's instructions. This involved impregnation of neural tissue with a potassium dichromate and silver nitrate solution to facilitate the precipitation of silver chromate onto a subset of neurons, rendering their entire dendritic and axonal arbors visible in their entirety. High‐resolution digital images of Golgi‐impregnated sections were captured using a Zeiss Axio Imager A2 microscope equipped with a high‐precision motorized stage and an AxioCam MRc digital camera. Quantitative morphological analyses were performed by an experimenter blinded to the treatment groups. For Golgi‐impregnated neurons, dendritic complexity was assessed using Sholl analysis. This involved placing concentric circles at 10 µm intervals radiating from the neuronal soma and counting the number of dendritic intersections with each circle. The data was used to generate Sholl plots and calculate the total dendritic length and branching complexity. Furthermore, dendritic spine density and morphological classification (e.g., stubby, thin, mushroom) were performed on selected secondary and tertiary apical dendritic branches (minimum 10 µm in length) at 100x magnification using SlideViewer v2.5 software (3D HISTECH).

### 4.5 Fecal Metagenomic Sequencing and Analysis

Fecal pellets from all mice within the same experimental group were pooled prior to DNA extraction and metagenomic library construction. Consequently, only a single aggregated metagenomic profile was generated per group. This pooling strategy precludes assessment of within‐group microbial variability and prohibits replicate‐dependent differential‐abundance analyses (e.g., LEfSe) and formal statistical testing of alpha‐diversity metrics. Fecal samples were collected and microbial genomic DNA was extracted. The quality and concentration of the extracted DNA were assessed. Qualified DNA samples were used for library construction. Sequencing was performed on an Illumina high‐throughput sequencing platform, generating paired‐end reads.

Raw sequencing data (in FASTQ format) were subjected to quality control. The process included: (1) removal of adapter sequences; (2) trimming of low‐quality reads, specifically those where the proportion of bases with a Phred quality score (Q‐score) ≤ 10 exceeded 50% of the read length, or where the proportion of ambiguous bases (N) exceeded 10%. The high‐quality reads obtained after this filtration are referred to as Clean Data. Quality metrics, including the percentage of bases with Q ≥ 20 and Q ≥ 30, GC content, and sequence length distribution, were calculated for each sample.

Quality‐controlled reads from all samples were co‐assembled *de novo* using MEGAHIT software, which is based on a de Bruijn graph algorithm [71]. Contigs with a length greater than 500 bp were retained for subsequent analysis. Open Reading Frame (ORF) prediction on the assembled contigs was performed using Prodigal software [72]. The predicted protein‐coding sequences (CDS) from all samples were clustered using CD‐HIT (with parameters set at 95% identity and 90% coverage) to generate a non‐redundant gene catalog [73]. The longest sequence within each cluster was selected as the representative gene sequence.

The construction of the gene abundance profile followed the standard metagenomic analysis pipeline [74]. Specifically, clean reads from each sample were aligned to a non‐redundant gene set using Bowtie2 [75] with a 95% identity threshold. The abundance of each gene in each sample was calculated in units of TPM (Transcripts Per Million) [76], which standardizes for both gene length and the total number of aligned reads per sample.

The predicted protein sequences from the non‐redundant gene catalog were aligned against the National Center for Biotechnology Information (NCBI) non‐redundant (NR) protein database (version 2021.11) using DIAMOND (BLASTP, e‐value ≤ 1e‐5) [77, 78]. The lowest common ancestor (LCA) algorithm, as implemented in MEGAN, was applied to assign taxonomic classifications (from kingdom to species level) to each gene [79, 80]. The abundance of a given taxon in a sample was defined as the sum of the abundances of all genes annotated to that taxon. Taxonomic composition was visualized using Krona plots [81], stacked bar charts, and clustering heatmaps [82]. Beta‐diversity was assessed using Bray‐Curtis distances [83] and visualized by PCoA, PCA, and Non‐metric Multidimensional Scaling (NMDS) [84, 85]. ANOSIM was not performed in the present study because only one pooled metagenomic profile was obtained per group, lacking independent biological replicates required for this test. Due to the pooled‐sample design without within‐group biological replicates, formal differential‐taxon‐abundance analyses including Metastat and LEfSe could not be applied to our metagenomic dataset.

Functional annotation was performed using DIAMOND [86] against the Kyoto Encyclopedia of Genes and Genomes (KEGG) [87], Non‐supervised Orthologous Groups (eggNOG) [88], and Carbohydrate‐Active enZYmes (CAZy) [89] databases. For KEGG, annotations were analyzed at multiple hierarchical levels (Pathway Levels 1–3, KO, and EC numbers). The relative functional abundance in a sample for a given pathway or category was calculated as the sum of the abundances of all genes annotated to that function. Functional composition was analyzed and visualized using stacked bar charts, heatmaps, and beta‐diversity ordination plots (PCoA, PCA, NMDS). Specific analyses of biogeochemical cycles (carbon, nitrogen, phosphorus, and sulfur) were conducted by extracting the abundance profiles of key functional marker genes from the KEGG annotation results.

Antibiotic resistance genes were identified by aligning the gene catalog against the Structured Antibiotic Resistance Gene (SARG) database (version 2.0) using its provided software (e‐value ≤ 1e‐5) [90]. Virulence factors were annotated by aligning against the core dataset of the Virulence Factor Database (VFDB) using DIAMOND (BLASTP, e‐value ≤ 1e‐5) [91]. The relative abundances of antibiotic resistance genes (ARGs) and virulence factors were calculated, and their distribution across samples was visualized using heatmaps and stacked bar charts. Species‐contribution analysis was performed to identify major microbial hosts for ARGs and virulence factors.

Metagenome‐assembled genomes (MAGs) were recovered from assembled contigs using VAMB [92]. The quality of MAGs was evaluated using CheckM [93], and those with > 50% completeness and <10% contamination were retained for downstream analysis [94]. Taxonomic classification was performed using GTDB‐Tk [95]. Relative abundances of MAGs across samples were also estimated.

### 4.6 Untargeted Plasma Metabolomics

Plasma samples were processed using a modified liquid‐liquid extraction protocol. Briefly, 100 µL of each plasma sample was transferred to a 1.5 mL microcentrifuge tube. A 400 µL aliquot of ice‐cold extraction solution (methanol:acetonitrile, 1:1, v/v) containing a mixture of deuterated internal standards was added. The mixture was vortexed vigorously for 30 s and then sonicated in an ice‐water bath for 10 min. To precipitate proteins, the samples were incubated at −40°C for 1 h. Subsequently, the samples were centrifuged at 12 000 rpm (RCF = 13 800 × *g*, radius = 8.6 cm) for 15 min at 4°C. The resulting supernatant was carefully collected and transferred into fresh glass vials for liquid chromatography with tandem mass spectrometry (LC‐MS/MS) analysis. A quality control (QC) sample was prepared by pooling equal volumes of the supernatant from all experimental samples.

Untargeted metabolomic profiling was performed using an ultra‐high‐performance liquid chromatography system (Vanquish, Thermo Fisher Scientific) coupled to an Orbitrap Exploris 120 mass spectrometer (Thermo Fisher Scientific). Chromatographic separation was achieved on a Waters ACQUITY UPLC BEH Amide column (2.1 mm × 50 mm, 1.7 µm particle size). The column temperature was maintained at 4°C, and the injection volume was 2 µL. The mobile phase consisted of 25 mmol/L ammonium acetate and 25 mmol/L ammonium hydroxide in water (solvent A, pH = 9.75) and pure acetonitrile (solvent B). Data acquisition was conducted in both positive (POS) and negative (NEG) ionization modes to maximize metabolite coverage. The mass spectrometer was operated in information‐dependent acquisition (IDA) mode controlled by Xcalibur software (v4.4, Thermo Fisher Scientific). The key electrospray ionization (ESI) parameters were: sheath gas flow rate, 50 Arb; auxiliary gas flow rate, 15 Arb; capillary temperature, 320°C; spray voltage, 3.8 kV (positive) or −3.4 kV (negative). Full MS scans were acquired at a resolution of 60 000, and MS/MS scans were triggered at a resolution of 15 000 with stepped normalized collision energies (SNCE) of 20, 30, and 40 eV.

Raw data files (.raw) were converted to the mzXML format using ProteoWizard software. Peak detection, alignment, and integration were performed using an in‐house R package based on the XCMS algorithm. Metabolite annotation was conducted by matching experimental data against a comprehensive in‐house database containing information on metabolite retention time (RT), precursor ion mass‐to‐charge ratio (MS1), and MS/MS fragmentation spectra (MS2). Annotation confidence levels were assigned according to the Metabolomics Standards Initiative (MSI) guidelines: Level 1, matched with an authentic standard in RT, MS1, and MS2; Level 2, matched with spectral data in a public database; Level 3, putatively characterized compound class; Level 4, unknown compound.

Following feature extraction, the data matrix was subjected to a series of pretreatment steps to ensure analytical quality. Features with a relative standard deviation (RSD) > 30% across QC samples were filtered out as noise. Additionally, features with more than 50% missing values in any single group or across all samples were removed. Remaining missing values were imputed with half of the minimum value for the corresponding feature. Normalization was performed using internal standard (IS) correction. After pretreatment, multivariate statistical analyses, including principal component analysis (PCA) and orthogonal projections to latent structures‐discriminant analysis (OPLS‐DA) [96], were performed using SIMCA‐P software (v18.0.1, Sartorius). The OPLS‐DA model was validated by 7‐fold cross‐validation and a 200‐time permutation test to prevent overfitting. Significantly altered metabolites were identified based on a variable importance in projection (VIP) score > 1.0 from the OPLS‐DA model and a *P*‐value < 0.05 from Student's t‐test. Pathway analysis and enrichment of differential metabolites were performed using the Kyoto Encyclopedia of Genes and Genomes (KEGG) [97] database and MetaboAnalyst 5.0 [98].

### 4.7 Single‐Cell RNA Sequencing (scRNA‐seq)

Hippocampal tissues from all mice within the same experimental group were pooled prior to tissue dissociation and single‐cell RNA sequencing (scRNA‐seq) library preparation. Consequently, only one aggregated scRNA‐seq dataset was generated per group. This pooling strategy precludes individual animal‐level paired multi‐omics correlation and network modeling analyses. Fresh tissues were dissociated into single‐cell suspensions following the manufacturer's protocol and the service provider's guidelines (Novogene Co., Ltd.). Cell viability and concentration were assessed before library construction, and only samples meeting the following quality control (QC) criteria were processed: cell viability > 80% (AO/PI staining) or > 75% (Trypan Blue staining), cell concentration between 700 and 1200 cells/µL, and a total cell count of up to 100 000.

Single‐cell RNA‐seq libraries were constructed using the 10x Genomics Chromium Single Cell 3′ Reagent Kit v3 (Dual Index) according to the manufacturer's instructions. Briefly, single‐cell suspensions, gel beads containing barcoded oligonucleotides, and partitioning oil were combined to generate Gel Bead‐In‐Emulsions (GEMs). Within each GEM, reverse transcription was performed to barcode cDNA from individual cells. The resulting barcoded cDNA was purified, amplified, and used for library construction. Final libraries were quantified and sequenced on an Illumina NovaSeq 6000 platform using a paired‐end 150 bp (PE150) strategy, aiming for a target sequencing depth of 50 000–100 000 reads per cell.

Raw sequencing data (FASTQ files) were processed using the Cell Ranger pipeline (v7.0.0, 10x Genomics). Briefly, cellranger mkfastq was used for demultiplexing. Then, cellranger count was used to align reads to the reference genome (species and genome version should be specified here, e.g., GRCm38/mm10 for mouse) using the STAR aligner, perform barcode and UMI counting, and generate the filtered gene‐barcode matrices. Cell calling was performed using the Cell Ranger algorithm, which distinguishes cell‐containing barcodes from background barcodes. The sample demultiplexing statistics, including estimated number of cells, mean reads per cell, median genes per cell, sequencing saturation, and mapping rates, are summarized in Table S6.

Downstream analysis was performed using the Seurat R package (v4.3.0) [99]. Cells were filtered based on three metrics [100]: (1) the number of detected genes per cell (nFeature_RNA), retaining cells with gene counts between 200 and 6000; (2) the percentage of mitochondrial gene counts (percent.mito), with a threshold of 25%–30%; and (3) the percentage of hemoglobin gene counts (percent.HB), with a threshold of 5%. Potential doublets (multiplets) were predicted and removed using the DoubletFinder package [101]. After filtering, gene expression counts were normalized using the LogNormalize method with a scale factor of 10 000, and the data were log‐transformed [102]. The 2000 most variable genes were identified using the FindVariableFeatures function. Data scaling was performed to regress out unwanted sources of variation (e.g., mitochondrial percentage) [103]. Principal component analysis (PCA) [104] was conducted on the scaled data of variable genes. Significant principal components (PCs) were selected for downstream nonlinear dimensionality reduction and clustering. Cells were clustered using a graph‐based clustering approach (Louvain algorithm) [105] on a K‐nearest neighbor (KNN) graph constructed in the PCA space. Cell clusters were visualized in two dimensions using t‐distributed stochastic neighbor embedding (t‐SNE) [106] and Uniform Manifold Approximation and Projection (UMAP) [107].

To identify marker genes for each cluster, differential expression analysis was performed using the Wilcoxon rank sum test [108] implemented in the Seurat FindAllMarkers function [109]. For each cluster, genes with an average log2 fold change > 0.25 and an adjusted *P*‐value < 0.05 were considered significantly upregulated [110]. Gene Ontology (GO) [111] and Kyoto Encyclopedia of Genes and Genomes (KEGG) [87] pathway enrichment analyses were performed on the marker gene sets of each cluster using the clusterProfiler R package (v3.14.0) [110]. Terms with a Benjamini‐Hochberg adjusted *P*‐value (padj) < 0.05 were considered significantly enriched [112]. Results were visualized using dot plots and bar plots [113].

### 4.8 Statistical Analysis

All data were tested for normality and homogeneity of variance before analysis. Parametric tests (ANOVA, Student's t‐test) or non‐parametric equivalents (Kruskal‐Wallis, Mann‐Whitney U) were applied as appropriate. Post hoc multiple comparisons were corrected using Tukey's or Benjamini‐Hochberg method. Correlations were assessed using Spearman's rank correlation coefficient. Data visualization was performed in GraphPad Prism (v9.0) and R. A *p*‐value < 0.05 was considered statistically significant.

## Author Contributions

Y.L., Y.P., and X.W. conceived the project and designed the experiments. Y.L., A.W., H.Z., and Y.S. performed the majority of the experiments and data analysis. Y.G., K.C., J.S., T.L., Z.H., X.Q., J.H., J.L., Y.Y., and S.S. assisted with data collection and technical support. Y.L., Y.P., and X.W. wrote the manuscript with input from all authors. Y.L., Y.P., and X.W. supervised the project.

## Declaration of Generative AI and AI‐Assisted Technologies in the Writing Process

Statement: During the preparation of this work, the authors used Deepseek‐R1 solely for the purpose of language refinement and polishing. After using this tool, the authors thoroughly reviewed and edited the output. The authors take full responsibility for the entire content of this published article, including its intellectual substance and accuracy.

## Funding

This work was funded by the National Natural Science Foundation of China (82472291 and 82372329), Zhejiang Provincial Natural Science Foundation of China (LTGD24C010001), Zhejiang Province key research and development plan project (2023C03048), Key Project of Health Science and Technology plan of Hangzhou (TD2023009), and Huzhou Municipal Science and Technology Bureau (2025YZ33, 2024GZ74, and 2024GZ81).

## Conflicts of Interest

The authors declare no conflicts of interest.

## Supporting information




**Supporting File 1**: advs78686‐sup‐0001‐SuppMat.pdf.

**Supporting File 2**: advs78686‐sup‐0002‐TableS1‐S15.zip.

## Data Availability

The data that support the findings of this study are available from the corresponding author upon reasonable request.
