# Supplementary material for: Bacillus Licheniformis‐Induced Cognitive Decline in Mice Linking Gut Microbiota Dysbiosis, Metabolic Perturbations, and Hippocampal Neuroinflammation
Source: Adv Sci (Weinh). 2026 Nov 9:e78686. Online ahead of print. doi: 10.1002/advs.78686 (PMC13748769; doi:10.1002/advs.78686)
Supplement: Supplementary file 1 [file ADVS-9999-e78686-s001.pdf]

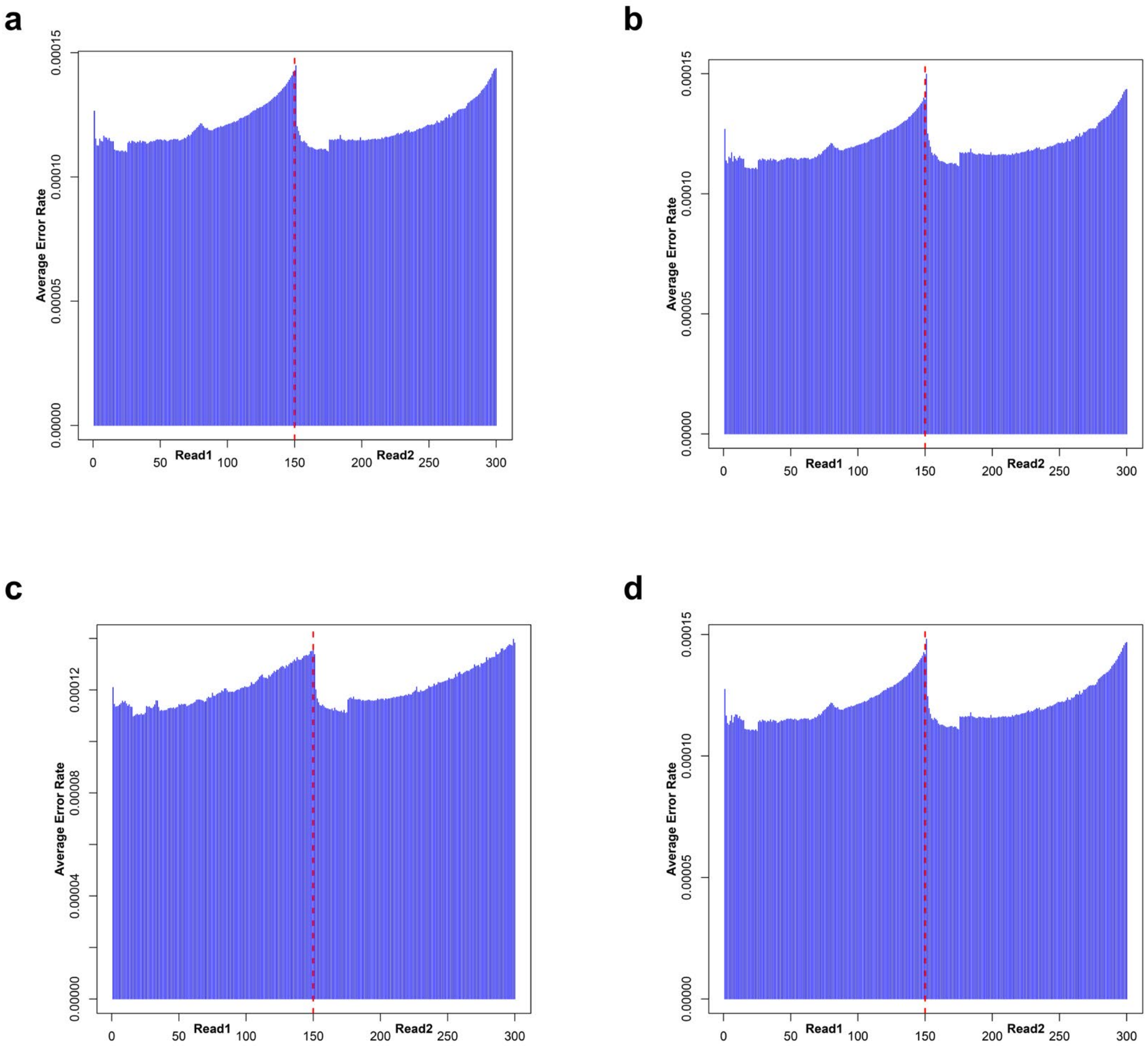

Supplementary Figure S1. Distribution map of base sequencing error rates. The horizontal axis represents the base position of the reads, and the vertical axis represents the single-base error rate. The red dashed line indicates the boundary between Read1 and Read2. (a) Control group; (b) *Bacillus licheniformis* intervention group; (c) *Clostridium butyricum* intervention group; (d) *Lactobacillus acidophilus* intervention group.

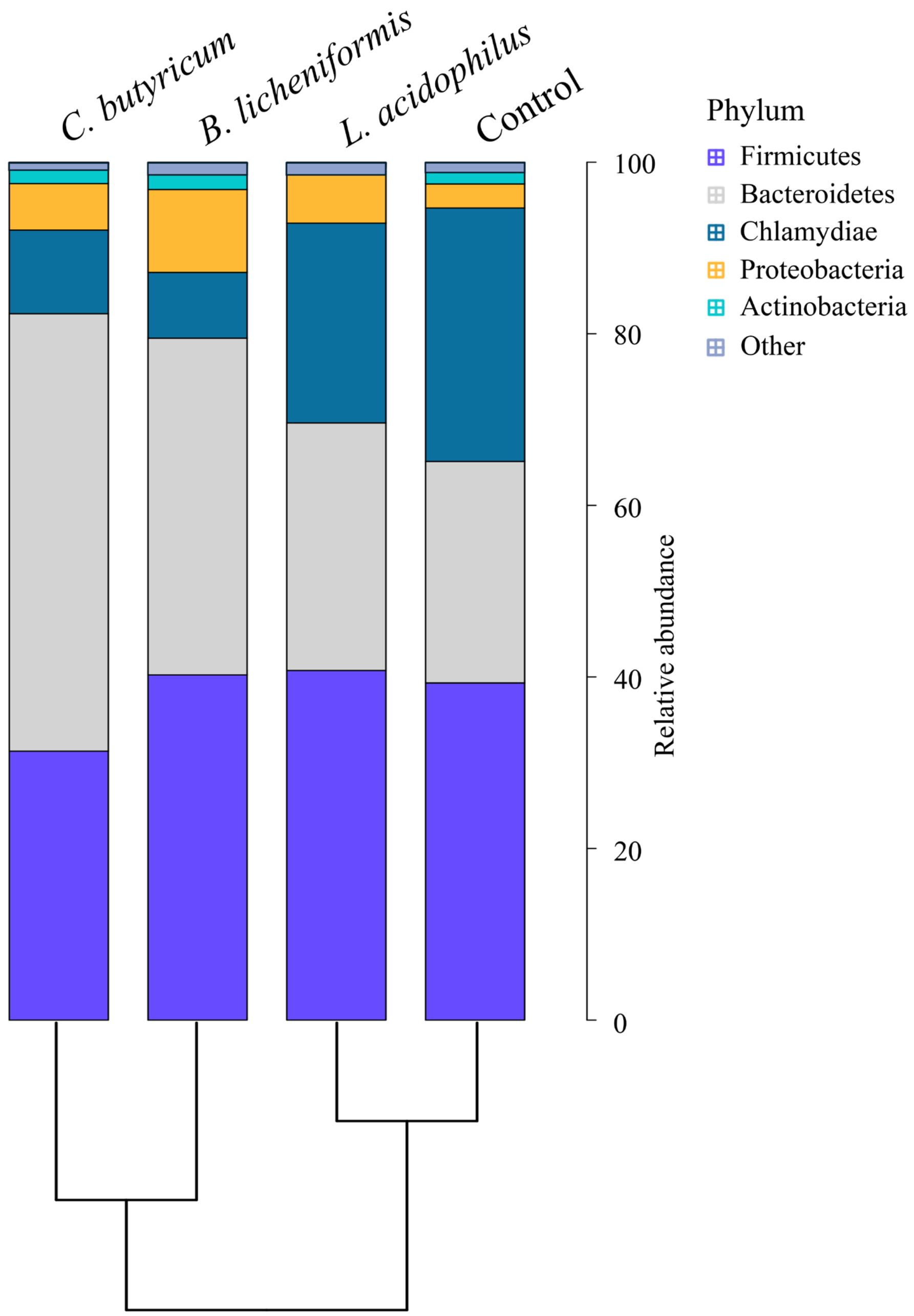

Supplementary Figure S2. Alterations in gut microbiota composition of mice after probiotic intervention. Stacked bar chart showing species abundance clustering and composition at the phylum level.

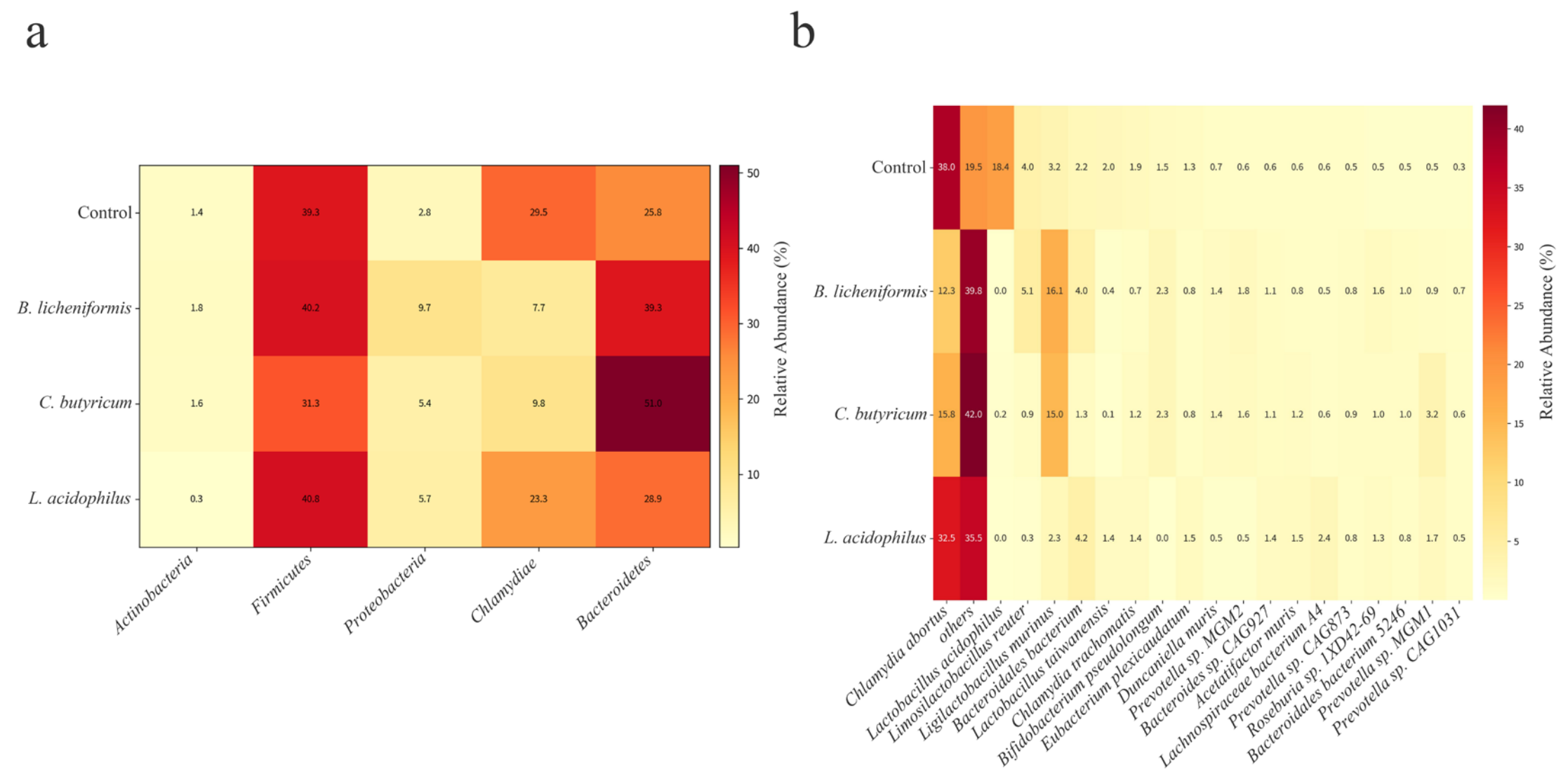

Supplementary Figure S3. Heatmap of the relative abundance of major microorganisms in different treatment groups. (a) Phylum level. The heatmap illustrates the relative abundance of bacterial phyla, including Actinobacteria, Firmicutes, Proteobacteria, Chlamydiae, and Bacteroidetes, across four different treatment groups (Control, *B. licheniformis*, *C. butyricum*, and *L. acidophilus*). (b) Species level. This panel displays the relative abundance of specific bacterial species, such as *Chlamydia abortus*, *Lactobacillus acidophilus*, *Limosilactobacillus reuter*, *Lactobacillus murinus*, *Bifidobacterium pseudolongum*, *Eubacterium plexicaudatum*, *Duncaniella muris*, *Lachnospiraceae bacterium* A4, *Prevotella* sp. CAG927, *Roseburia* sp. 1XD42-69, *Acetatifactor muris*, *Prevotella* sp. MGM1, *Prevotella* sp. CAG1031, among others. The color scale on the right indicates the relative abundance percentage, with darker red representing higher abundance and lighter yellow representing lower abundance. The rows represent different bacterial taxa, and the columns represent different grouped averages. Hierarchical clustering was performed to group similar samples and taxa based on their abundance patterns.

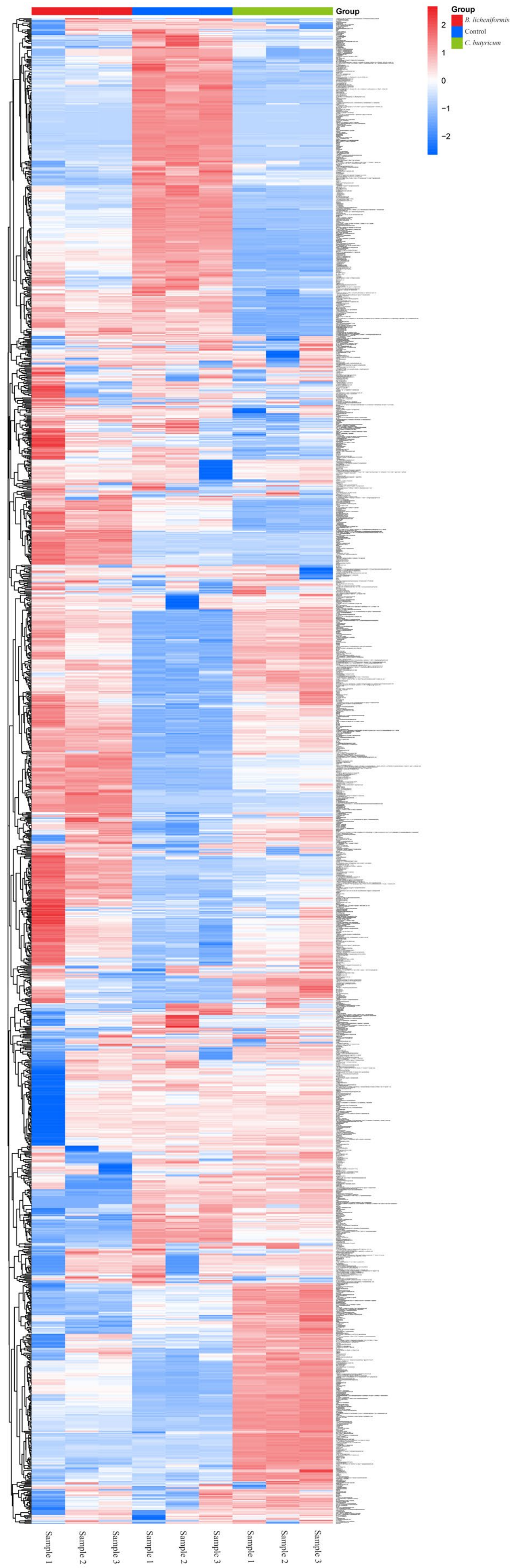
Group
Group
B. licheniformis
Control
C. butyricum
2
1
0
−1
−2
Sample 1
Sample 2
Sample 3
Sample 1
Sample 2
Sample 3
Sample 1
Sample 2
Sample 3

Supplementary Figure S4. TOTAL Serum metabolite heatmap. Clustered heatmap displaying the relative abundance of serum metabolites across all samples (labeled individually on the x-axis). The samples were grouped based on the intervention: Control (blue), *B. licheniformis* (red), and *C. butyricum* (green), as indicated by the color bar at the top. Metabolites (rows) and samples (columns) were hierarchically clustered using Ward's linkage algorithm. The color scale on the right indicates the standardized abundance values, where red represents higher abundance (up-regulation) and blue represents lower abundance (down-regulation) relative to the mean. The dendrograms on the left and at the top illustrate the hierarchical clustering relationships.

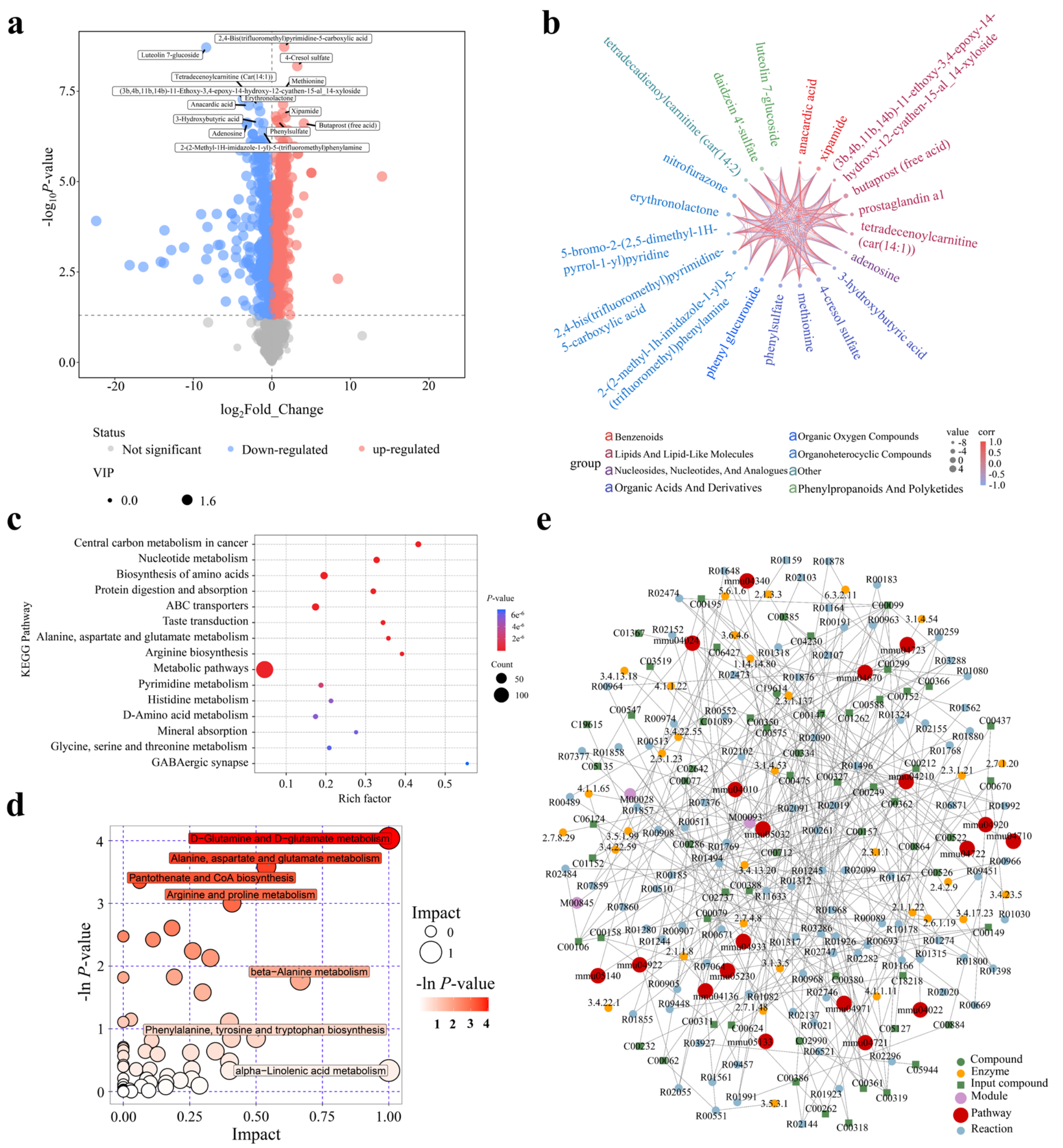

Supplementary Figure S5. Altered metabolites in serum of *C. butyricum* intervention in mice compared to control. (a) Volcano plot for differential metabolite screening between *C. butyricum* and control groups. Red and blue dots represent significantly up-regulated and down-regulated metabolites, respectively. Gray dots indicate non-significant metabolites. (b) Chord diagram illustrating the association between differential metabolites (labeled on the outer circle with color-coded metabolite classes) and KEGG pathways (inner labels). (c) KEGG pathway enrichment analysis presented as a bar plot. The x-axis represents the Rich Factor, and the size of the dots indicates the number of metabolites enriched in each pathway. (d) Pathway

impact analysis displayed as a scatter plot. The size of the circles represents the Impact value, and the color intensity indicates the statistical significance (-ln P-value). (e) Regulatory network analysis between differential metabolites and related genes/proteins. Nodes represent compounds (red), enzymes (yellow), genes (purple), pathways (brown), reactions (light blue), and proteins (dark blue), connected by edges indicating regulatory relationships.

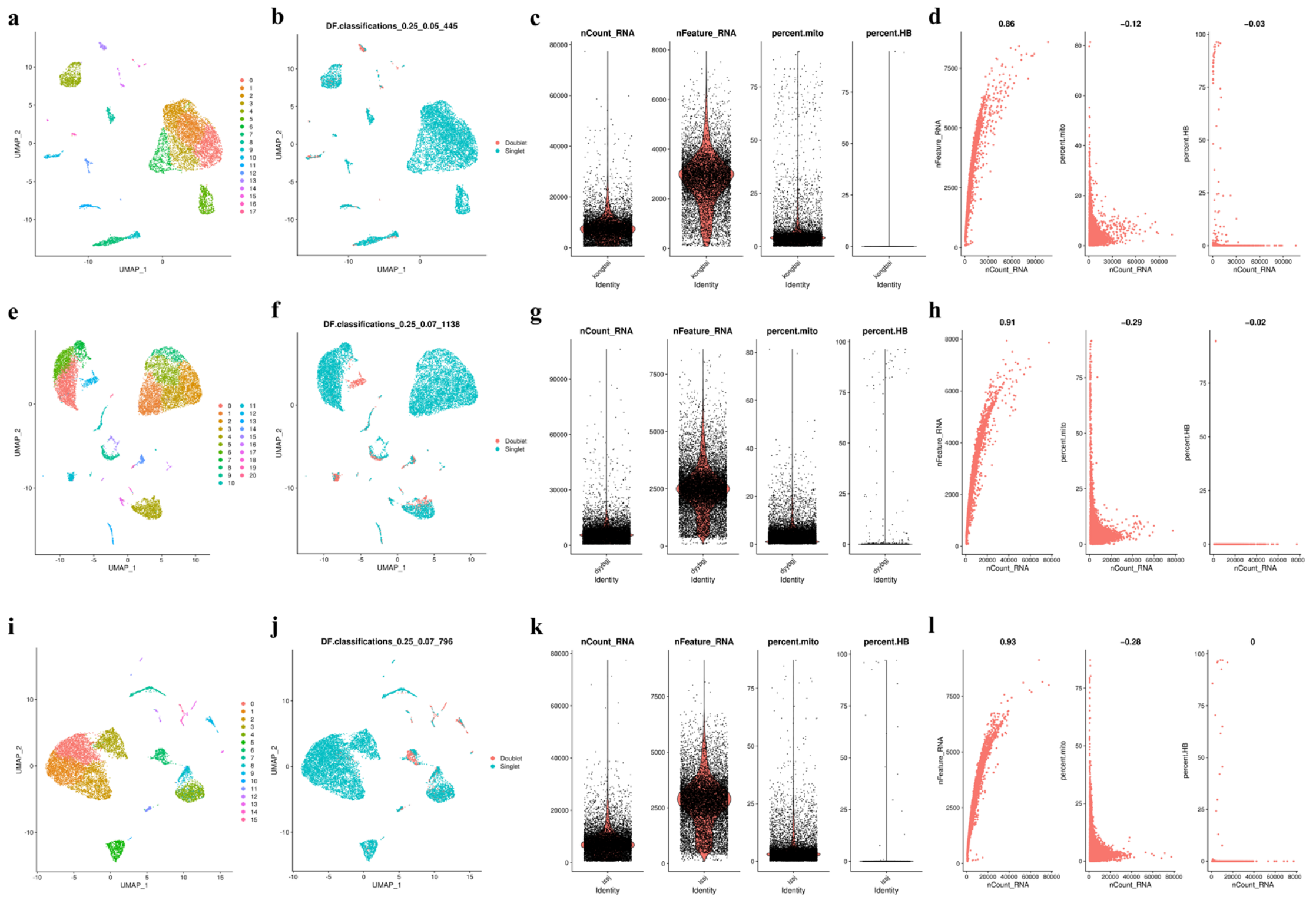

Supplementary Figure S6. Visual display of consequences from the given filtering conditions in cell. (a) cluster_UMAP, (b) cluster_UMAP_single_doublet, (c) nGene_nUMI_mito_HB, and (d) nGene_nUMI_mito_HB_relation for Control; (e) cluster_UMAP, (f) cluster_UMAP_single_doublet, (g) nGene_nUMI_mito_HB, and (h) nGene_nUMI_mito_HB_relation for *B. licheniformis* administrated group; (i) cluster_UMAP, (j) cluster_UMAP_single_doublet, (k) nGene_nUMI_mito_HB, and (l) nGene_nUMI_mito_HB_relation for *C. butyricum* administrated group. Cell filtering was performed using the packages Seurat and DoubletFinder. The clustering results shown here are only preliminary quality control visualizations, not the final clustering outcome. In the nGene_nUMI_mito_HB_relation plots: As a cell's nCount increases, the number of detected genes also tends to increase (Plot 1). High mitochondrial proportions or high hemoglobin gene proportions are observed mainly in cells with low nCount (Plots 2 and 3). nFeatures_RNA: Number of features (genes) detected per cell. percent.mito: Percentage of mitochondrial genes. percent.HB: Percentage of hemoglobin genes. UMAP: Uniform manifold approximation and projection. UMI: Unique molecular identifiers.

Notes:

cluster_UMAP: UMAP plot generated by Seurat without removing doublets, showing clustering results before multiplet removal.

cluster_UMAP_single_doublet: UMAP visualization after doublet detection. Doublet refers to multiplets identified by DoubletFinder.

nGene_nUMI_mito_HB: Distribution plots generated by Seurat for the proportions of nCount (UMI count), nFeature (number of detected genes), mitochondrial gene percentage, and hemoglobin (HB) percentage.

nGene_nUMI_mito_HB_relation: Plots generated by Seurat showing how nFeature (gene expression count), mito (mitochondrial gene expression proportion), and HB (hemoglobin gene proportion) change with increasing nCount. The numbers on the plots indicate the correlation coefficients between the two variables.

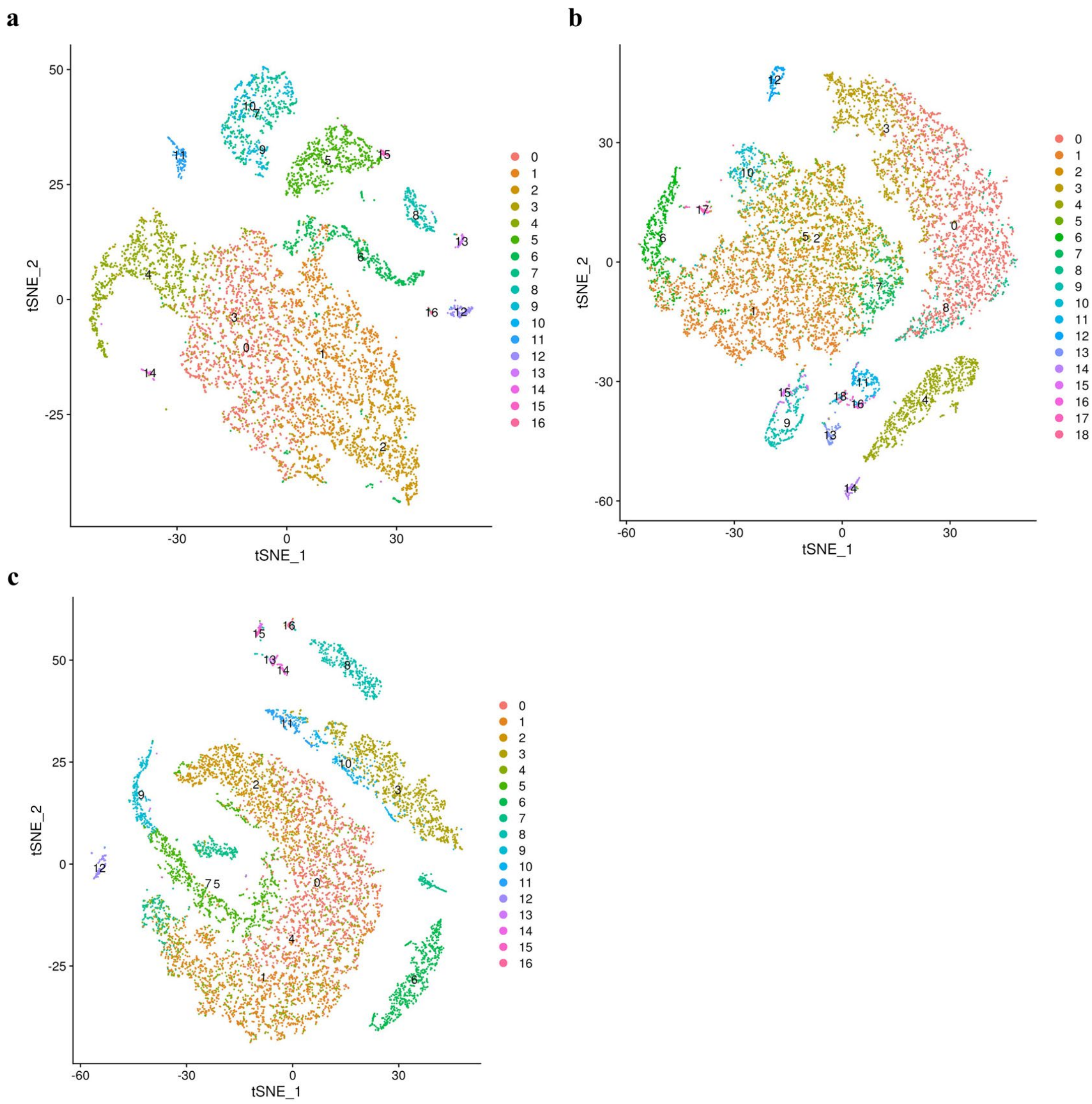

Supplementary Figure S7. Cell clustering analysis visualization via t-SNE after intervention with probiotics. (a) Control group. (b) *B. licheniformis* intervention group. (c) *C. butyricum* intervention group.

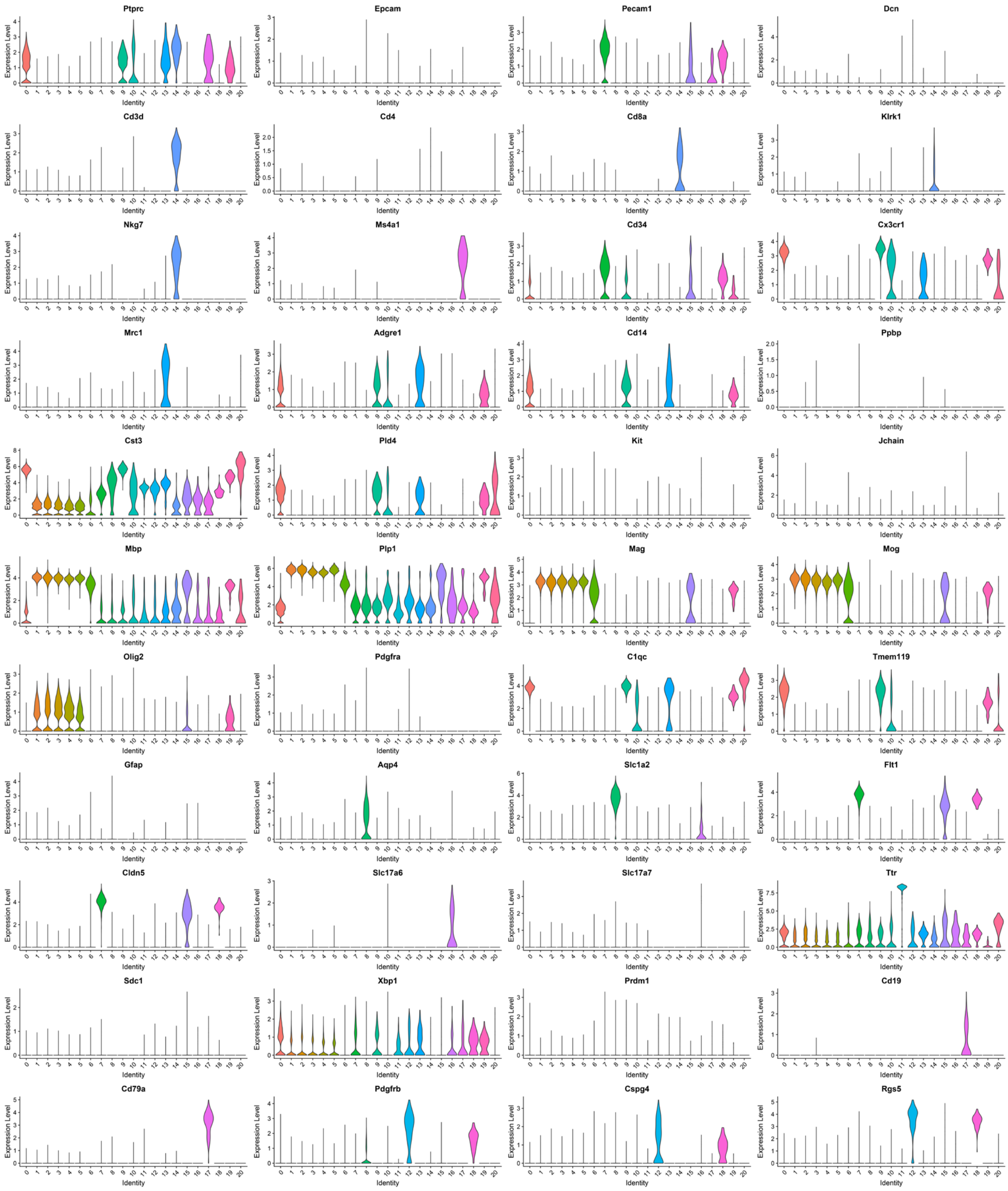

Supplementary Figure S8. Combined violin plots displaying the expression levels of canonical cell type-specific markers across identified clusters. The X-axis represents individual clusters (annotated with cell identities in some plots), while the Y-axis shows gene expression level (log-normalized). Each row highlights a specific set of marker genes: (1) *Ptprc*, *Epcam*, *Pecam1*, and *Dcn*; (2) *Cd3d*, *Cd4*, *Cd8a*, and *Klrk1*; (3) *Nkg7*,

Ms4a1, *Cd34*, and *Cx3cr1*; (4) *Mrc1*, *Adgre1*, *Cd14*, and *Ppbp*; (5) *Cst3*, *Pld4*, *Kit*, and *Jchain*; (6) *Mbp*, *Plp1*, *Mag*, and *Mog*; (7) *Olig2*, *Pdgfra*, *C1qc*, and *Tmem119*; (8) *Gfap*, *Aqp4*, *Slc1a2*, and *Flt1*; (9) *Cldn5*, *Slc17a6*, *Slc17a7*, and *Ttr*; (10) *Sdc1*, *Xbp1*, *Prdm1*, *Cd19*; (11) *Cd79a*, *Pdgfrb*, *Cspg4*, and *Rgs5*. Differential expression patterns reveal distinct cellular identities within the hippocampus, including endothelial cells (*Pecam1*, *Cldn5*), choroid plexus cells and choroid plexus epithelial cells (*Epcam*, *Ttr*), excitatory neurons (*Slc17a6*, *Slc17a7*), T cells (*Cd3d*, *Cd4*, *Cd8a*, *Klrk1*), B cells (*Ms4a1*, *Cd19*, *Cd79a*, *Jchain*, *Sdc1*), microglia (*Cx3cr1*, *C1qc*, *Tmem119*), oligodendrocytes (*Mbp*, *Plp1*, *Olig2*), astrocytes (*Gfap*, *Aqp4*, *Slc1a2*), and pericytes (*Pdgfrb*, *Cspg4*, *Rgs5*). Doublets, characterized by mixed expression profiles from two distinct cell types (e.g., *Ptprc*, *Cd34*), are also observed in specific clusters.

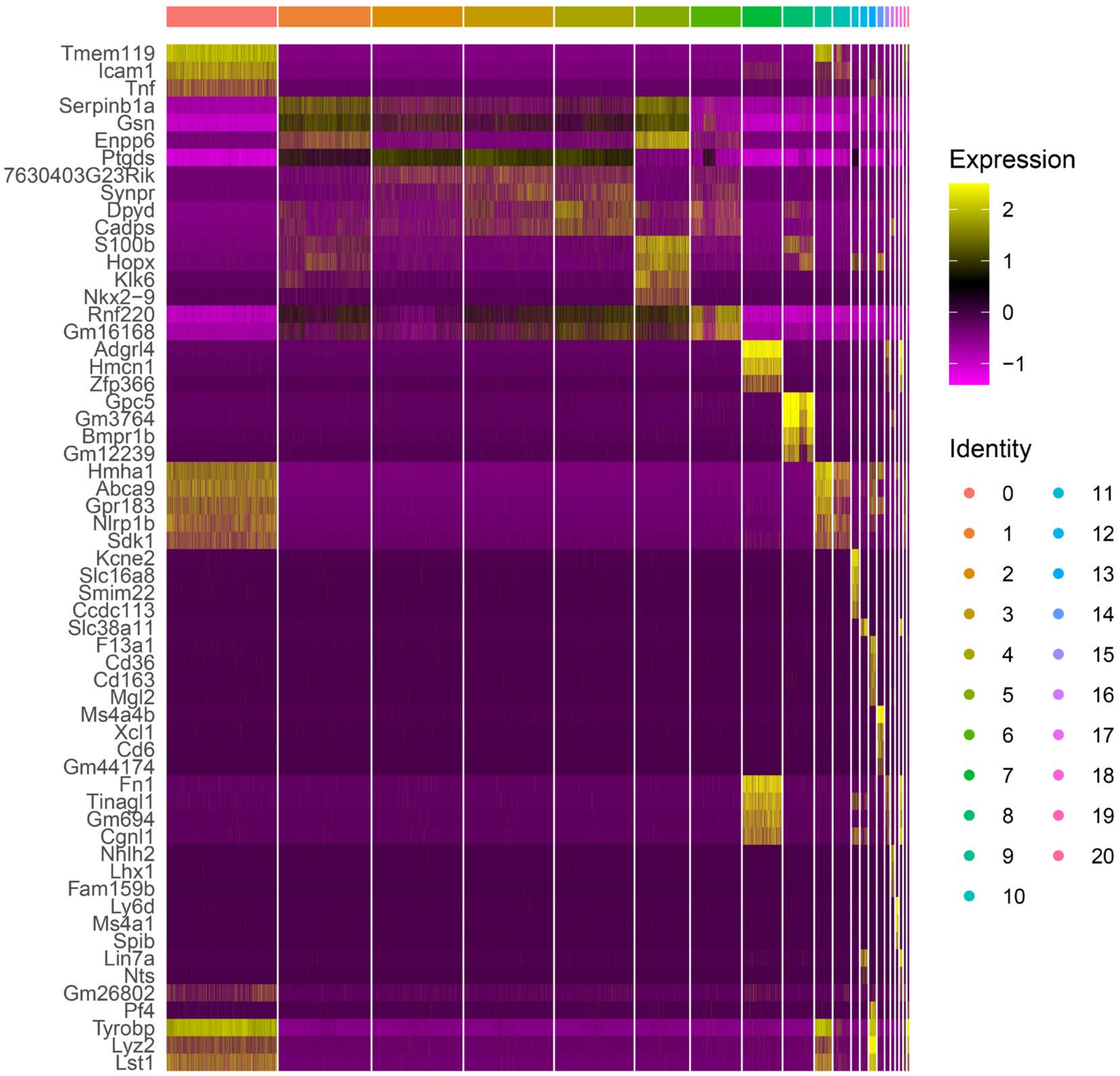

Supplementary Figure S9. Heatmap presenting the marker genes expression levels among hippocampal cell subpopulations. Clustered heatmap illustrating the expression levels of selected genes across different cell clusters. The rows represent individual genes (e.g., *Tmem119*, *Icam1*, *Tnf*, *Serpinb1a*), and the columns represent individual cells. Cells are grouped into 21 clusters (annotated as identities 0–20 on the right) based on their gene expression profiles, with each cluster indicated by a distinct color. The color scale on the right (ranging from purple to yellow) indicates the normalized gene expression level, where yellow represents higher expression and purple represents lower expression. The top color bar visually segregates the cell clusters for easier visualization of the grouped expression patterns.

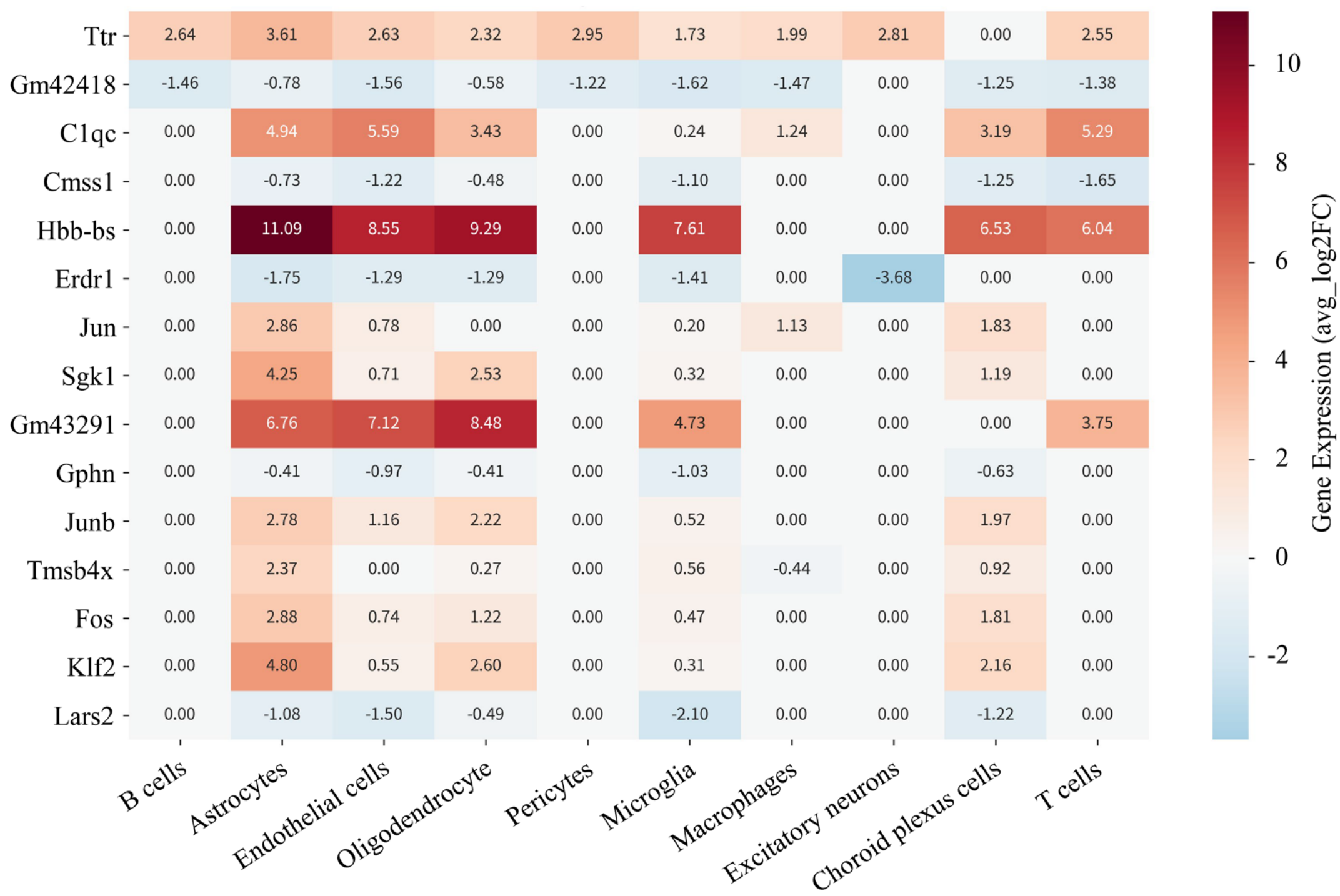

Supplementary Figure S10. Heatmap showing the log2 fold change of genes that are significantly expressed in multiple cell types. The heatmap illustrates the average log2 fold change (avg_log2FC) of specifically expressed genes across various cell types within the choroid plexus. The rows represent individual genes (e.g., *Ttr*, *Hbb-bs*, *Gm43291*, *C1qc*), while the columns represent different cell types, including B cells, Astrocytes, Endothelial cells, Oligodendrocyte, Pericytes, Microglia, Macrophages, Excitatory neurons, Choroid plexus cells, and T cells. The color scale on the right indicates the magnitude of gene expression change: red represents upregulation (positive values), blue represents downregulation (negative values), and white indicates negligible change (values close to 0). The numerical values within each cell denote the specific avg_log2FC for the corresponding gene in that cell type, providing a quantitative measure of differential expression. This visualization highlights the cellular specificity of gene expression programs, with distinct gene panels showing high expression in immune-related cells (e.g., *Hbb-bs* in Macrophages) or other specialized cell populations.

Notes:

avg_log2FC: Average log2 fold change in gene expression.

Color scale: Red = upregulated; Blue = downregulated; White = no significant change.

Genes are listed on the y-axis; cell types are labeled on the x-axis.

Values shown are specific to the displayed genes and cell types, with some cells left blank (0.00) indicating no significant differential expression detected.

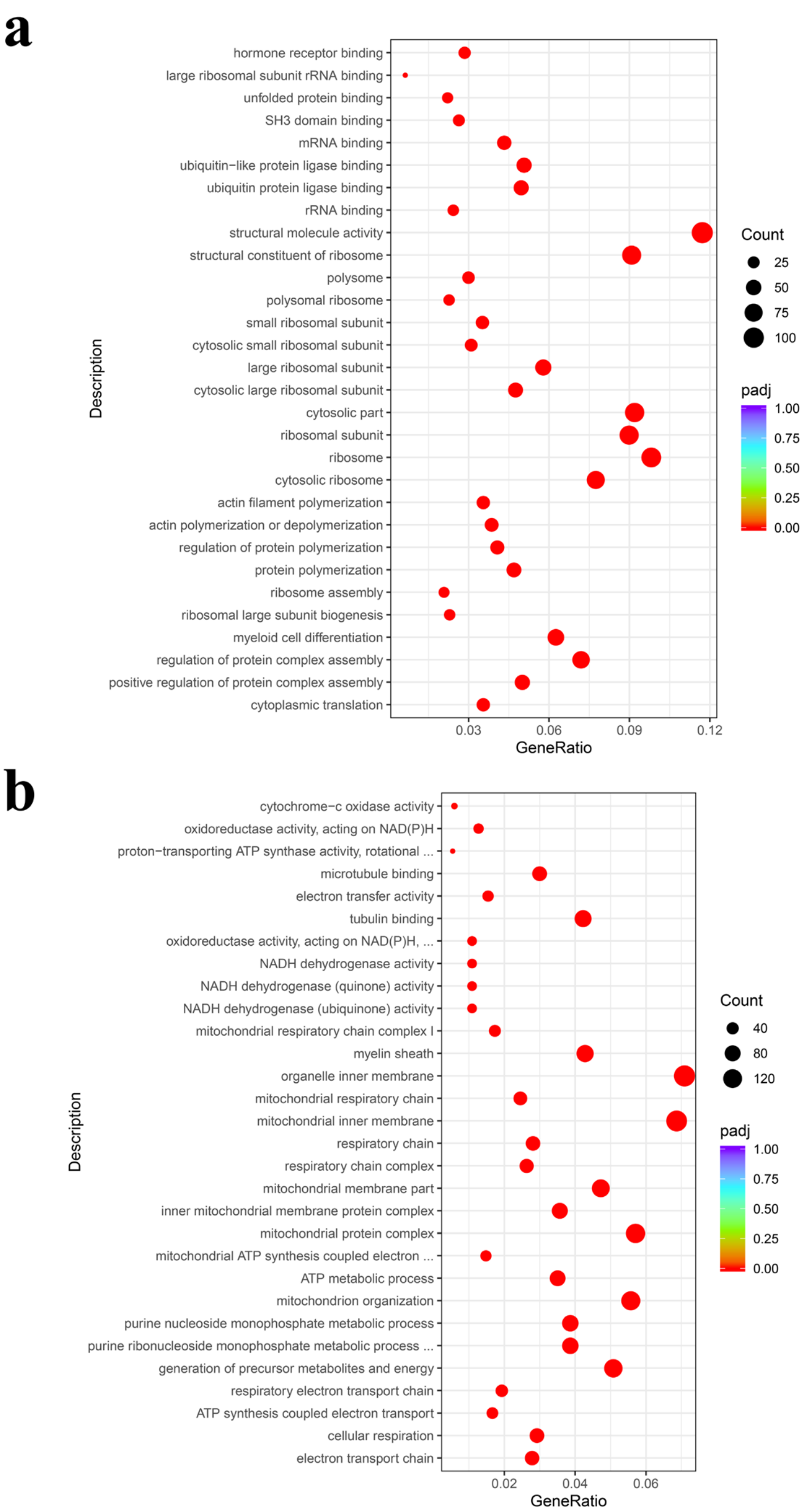

Supplementary Figure S11. Gene ontology functional enrichment analysis of up-regulated (a) and down-regulated (b) hippocampal differentially expressed genes (DEGs) following *B. licheniformis* intervention. The horizontal axis (GeneRatio) represents the ratio of the number of DEGs enriched in a specific biological process to the total number of DEGs. The size of the dots is proportional to the number of genes (Count) involved in each biological process. The color gradient indicates the adjusted P-value (padj), with red representing higher statistical significance (lower padj values).

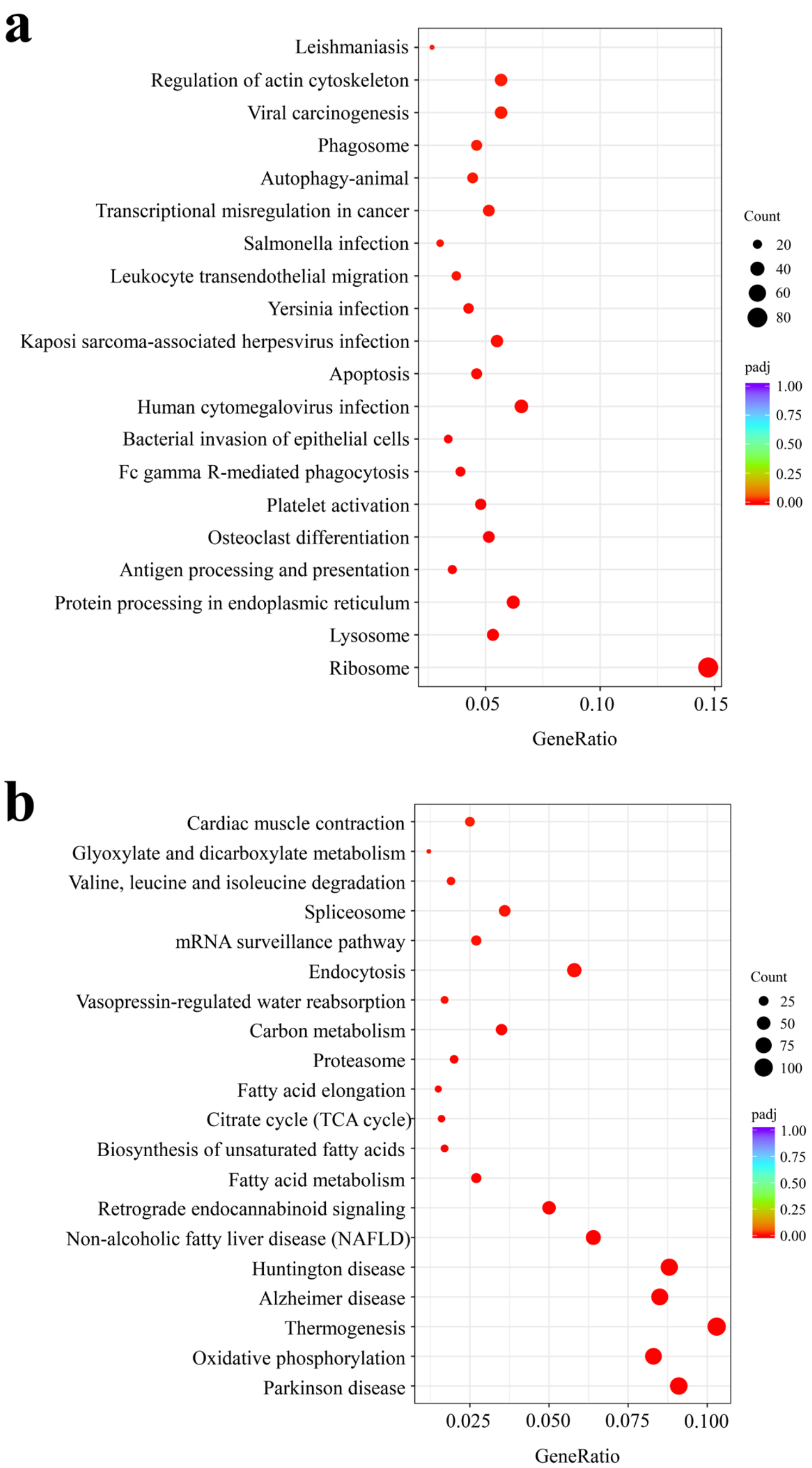

Supplementary Figure S12. KEGG functional enrichment analysis of up-regulated (a) and down-regulated (b) hippocampal differentially expressed genes (DEGs) following *B. licheniformis* intervention. The horizontal axis (GeneRatio) represents the ratio of the number of DEGs enriched in a specific pathway to the total number of DEGs. The size of the dots is proportional to the number of genes (Count) involved in each pathway. The color gradient indicates the adjusted p-value (padj), with red representing higher statistical significance (lower padj values).

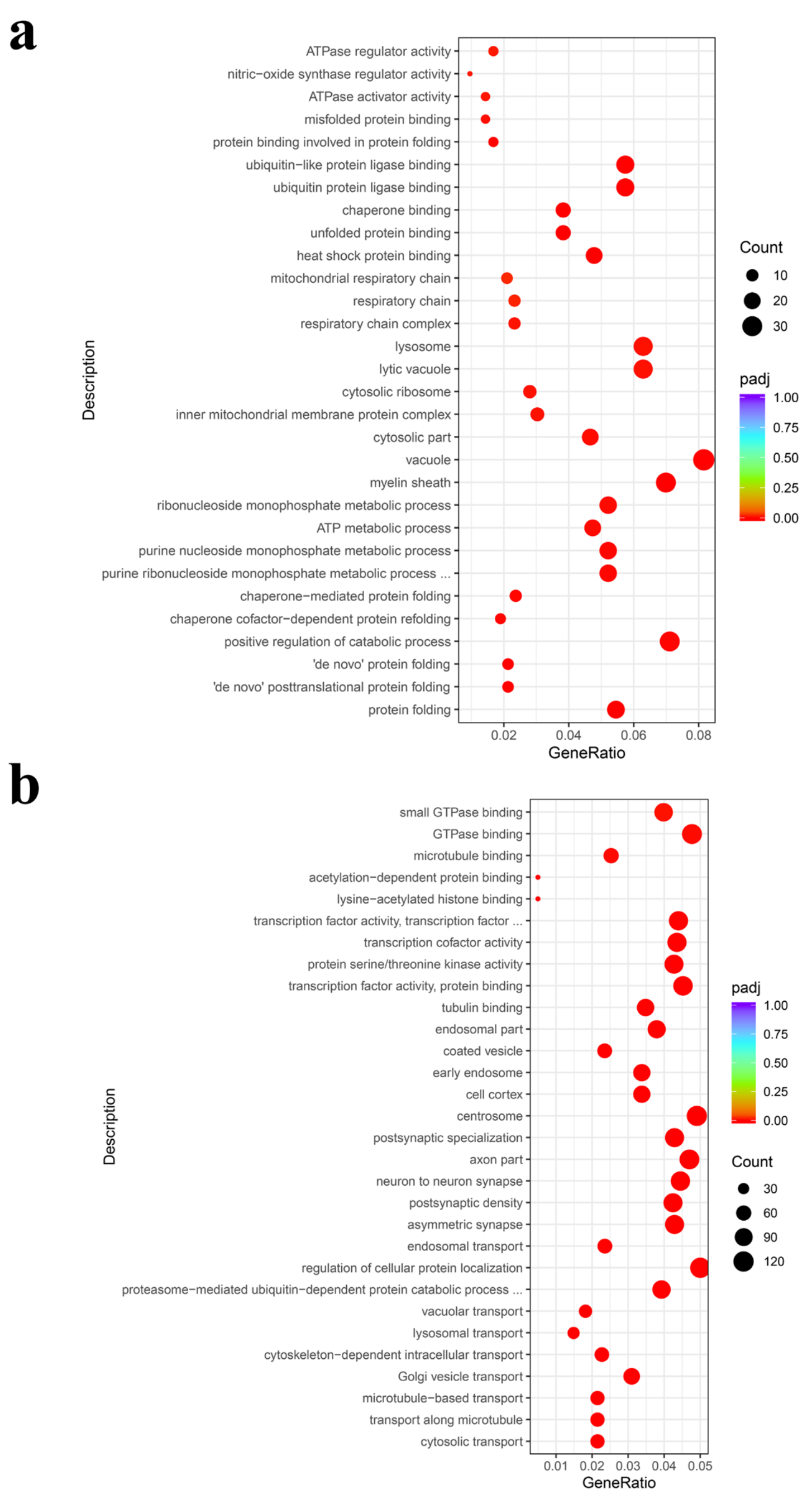

Supplementary Figure S13. Gene ontology functional enrichment analysis of up-regulated (a) and down-regulated (b) hippocampal DEGs in oligodendrocytes following *B. licheniformis* intervention. The horizontal axis (GeneRatio) represents the ratio of the number of DEGs enriched in a specific biological process to the total number of DEGs. The size of the dots is proportional to the number of genes (Count) involved in each biological process. The color gradient indicates the adjusted P-value (padj), with red representing higher statistical significance (lower padj values).

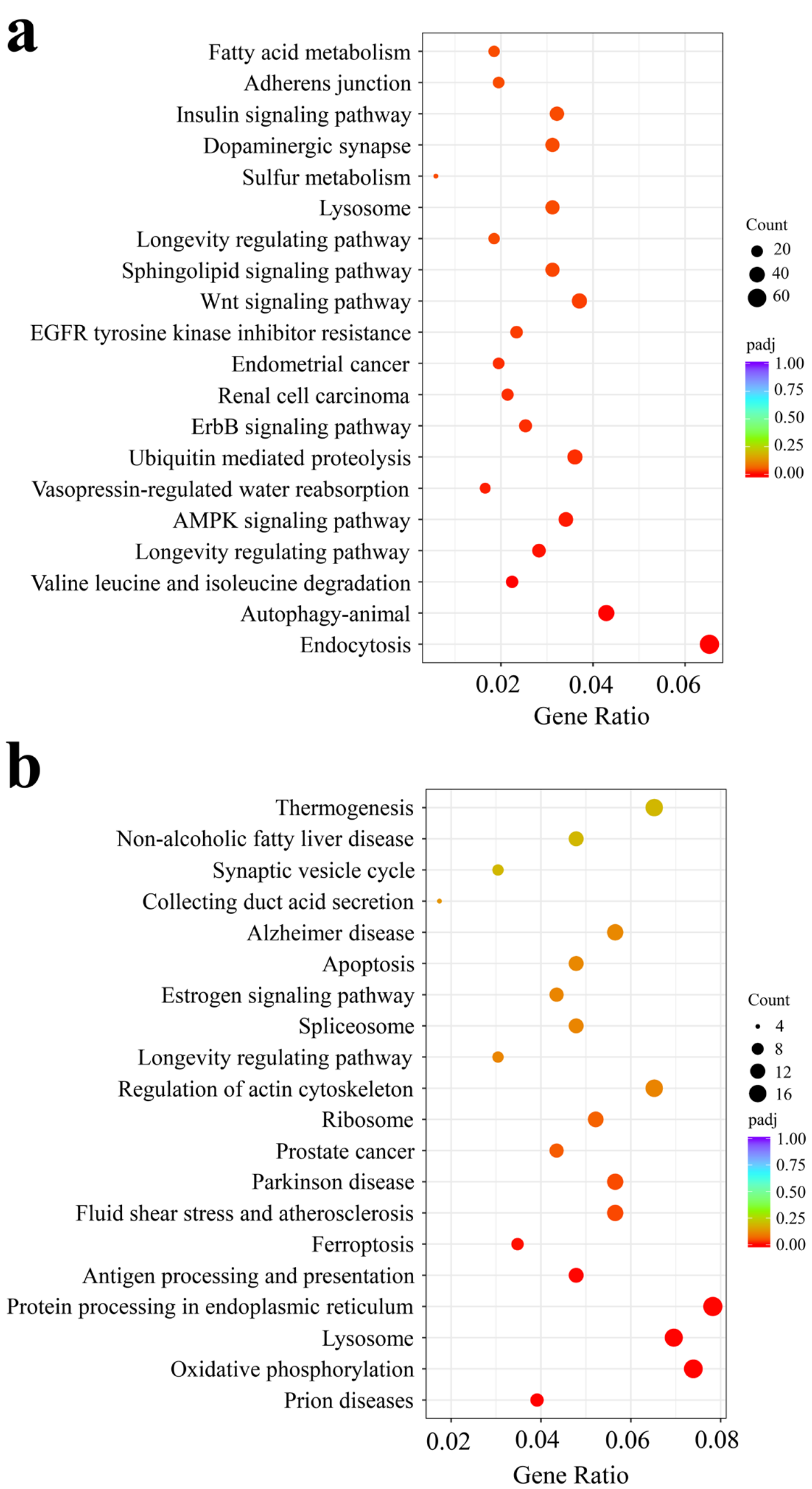

Supplementary Figure S14. KEGG functional enrichment analysis for genes that are up-regulated (a) or down-regulated (b) in oligodendrocytes following *B. licheniformis* intervention. The horizontal axis (GeneRatio) represents the ratio of the number of DEGs enriched in a specific pathway to the total number of DEGs. The size of the dots is proportional to the number of genes (Count) involved in each pathway. The color gradient indicates the adjusted p-value (padj), with red representing higher statistical significance (lower padj values).

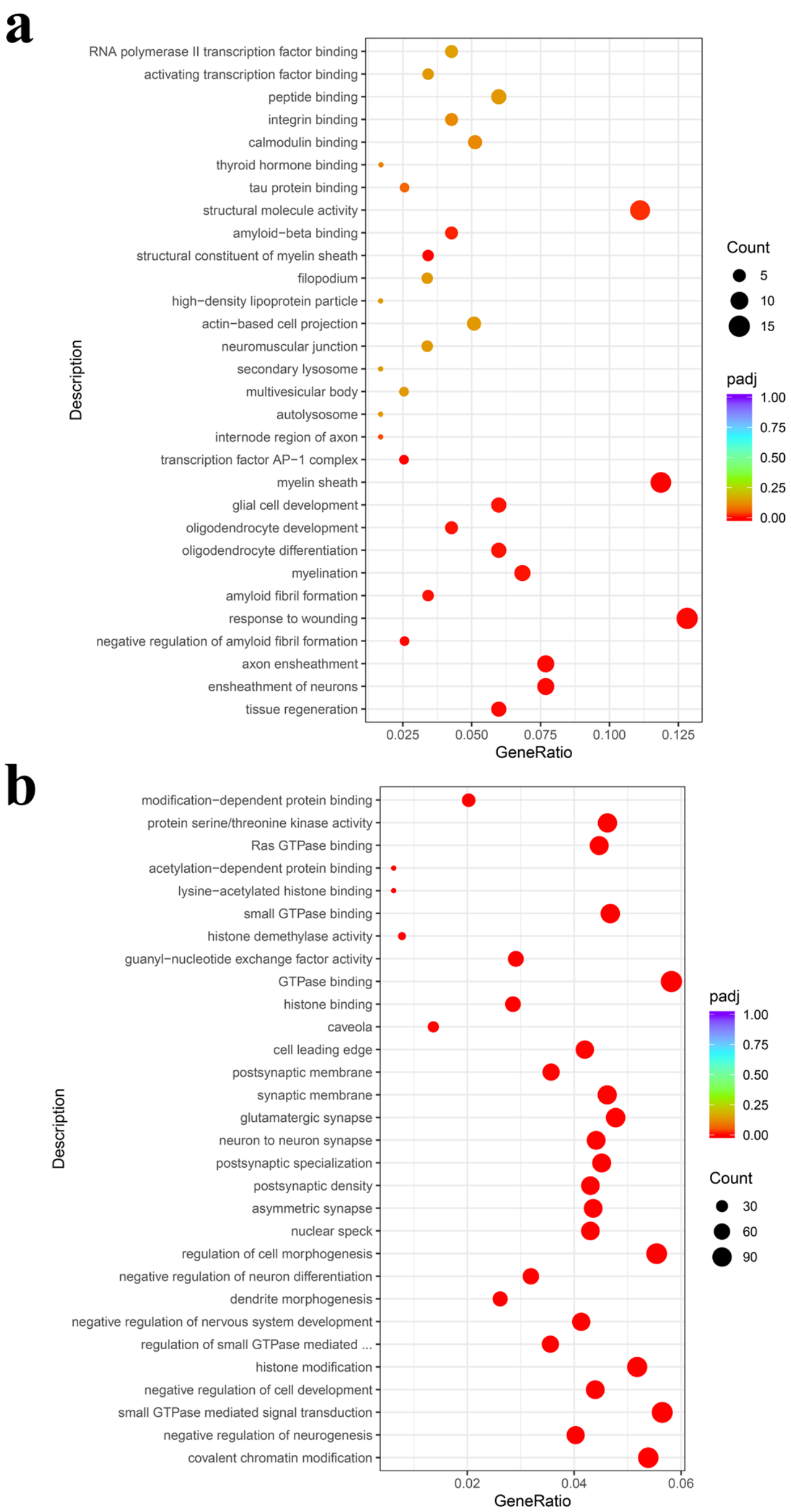

Supplementary Figure S15. Gene ontology functional enrichment analysis of up-regulated (a) and down-regulated (b) hippocampal DEGs in astrocytes following *B. licheniformis* intervention. The horizontal axis (GeneRatio) represents the ratio of the number of DEGs enriched in a specific biological process to the total number of DEGs. The size of the dots is proportional to the number of genes (Count) involved in each biological process. The color gradient indicates the adjusted P-value (padj), with red representing higher statistical significance (lower padj values).

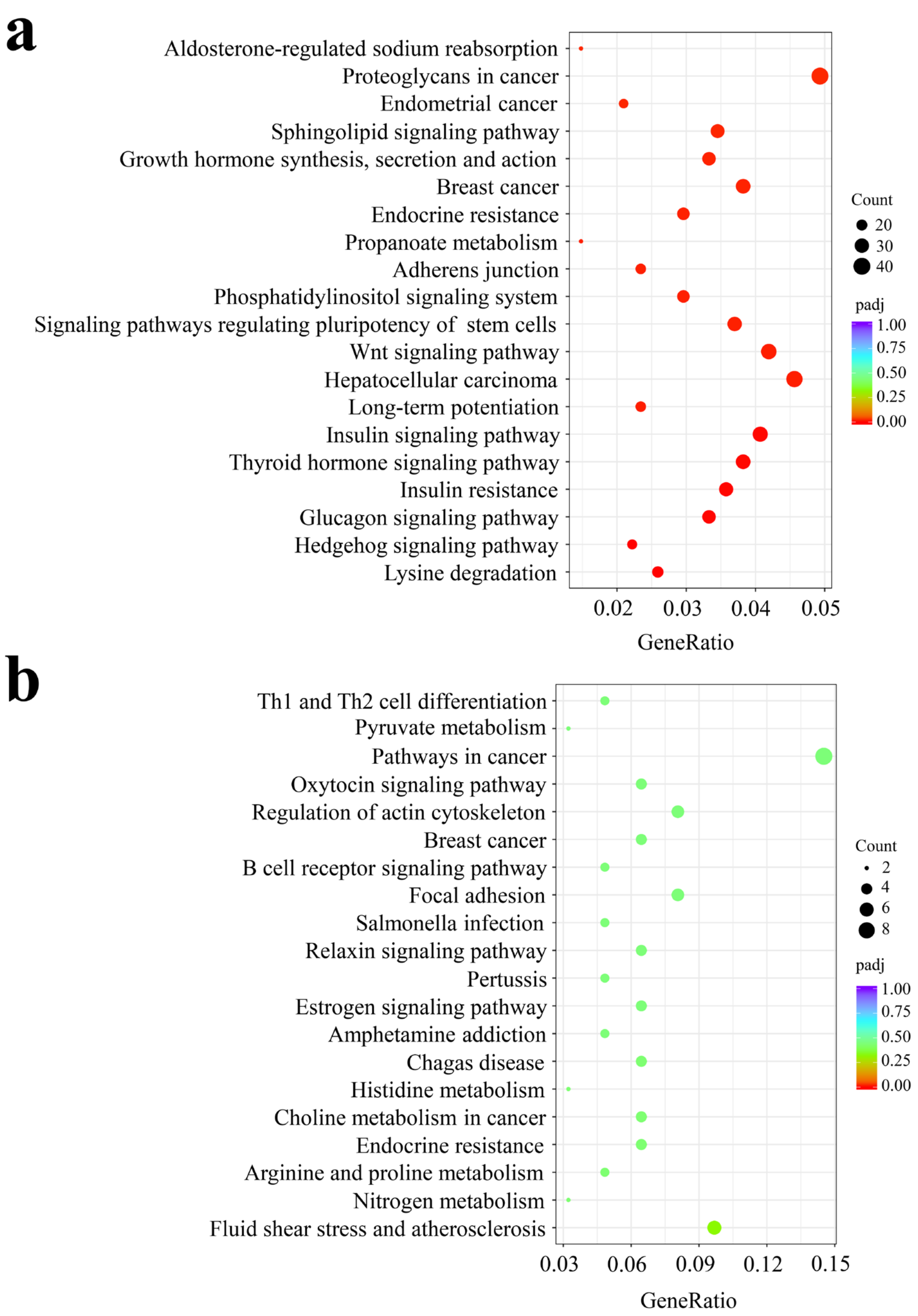

Supplementary Figure S16. KEGG functional enrichment analysis for genes that are up-regulated (a) or down-regulated (b) in astrocytes following *B. licheniformis* intervention. The horizontal axis (GeneRatio) represents the ratio of the number of DEGs enriched in a specific pathway to the total number of DEGs. The size of the dots is proportional to the number of genes (Count) involved in each pathway. The color gradient indicates the adjusted p-value (padj), with red representing higher statistical significance (lower padj values).

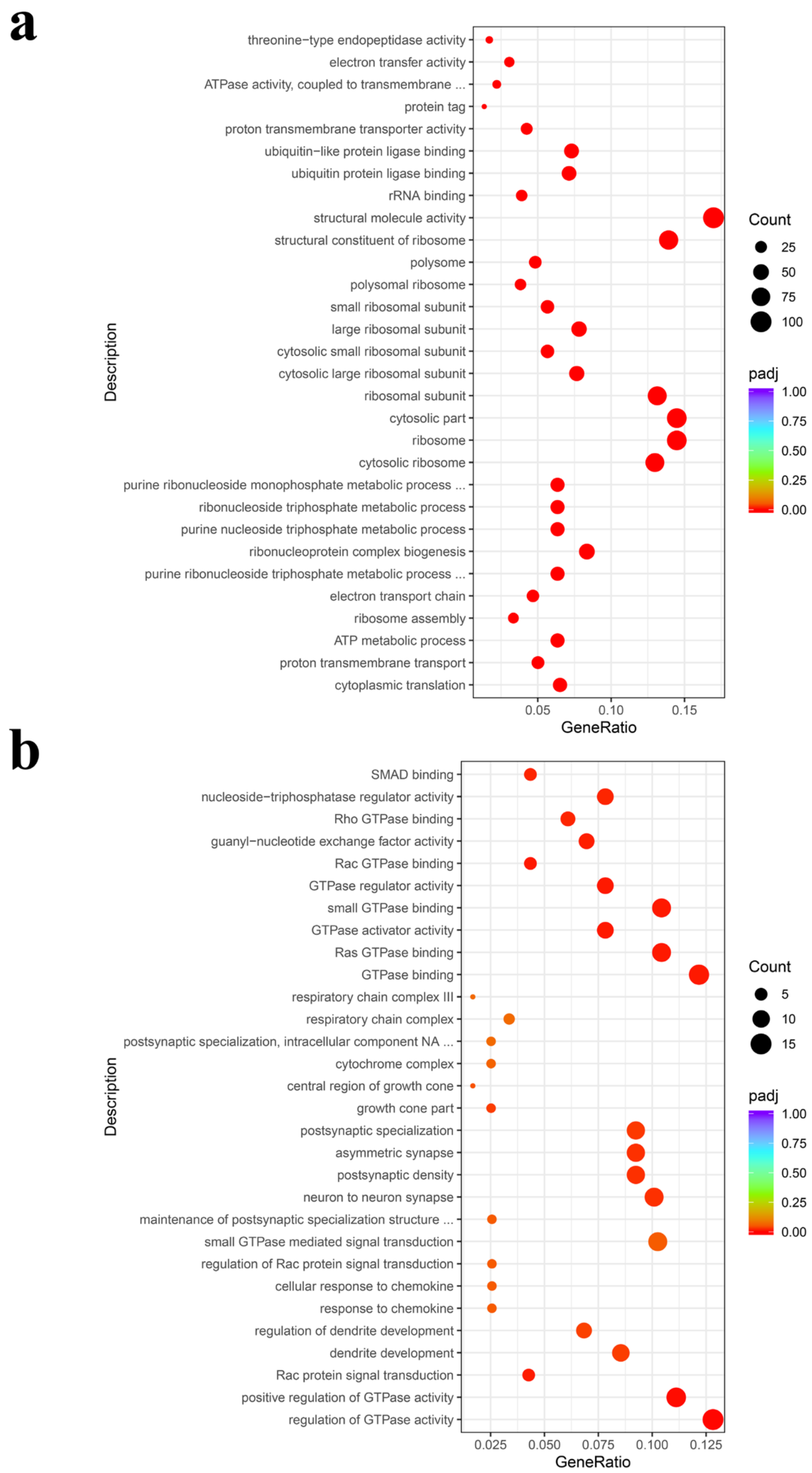

Supplementary Figure S17. Gene ontology functional enrichment analysis of up-regulated (a) and down-regulated (b) hippocampal DEGs in microglia following *B. licheniformis* intervention. The horizontal axis (GeneRatio) represents the ratio of the number of DEGs enriched in a specific biological process to the total number of DEGs. The size of the dots is proportional to the number of genes (Count) involved in each biological process. The color gradient indicates the adjusted P-value (padj), with red representing higher statistical significance (lower padj values).

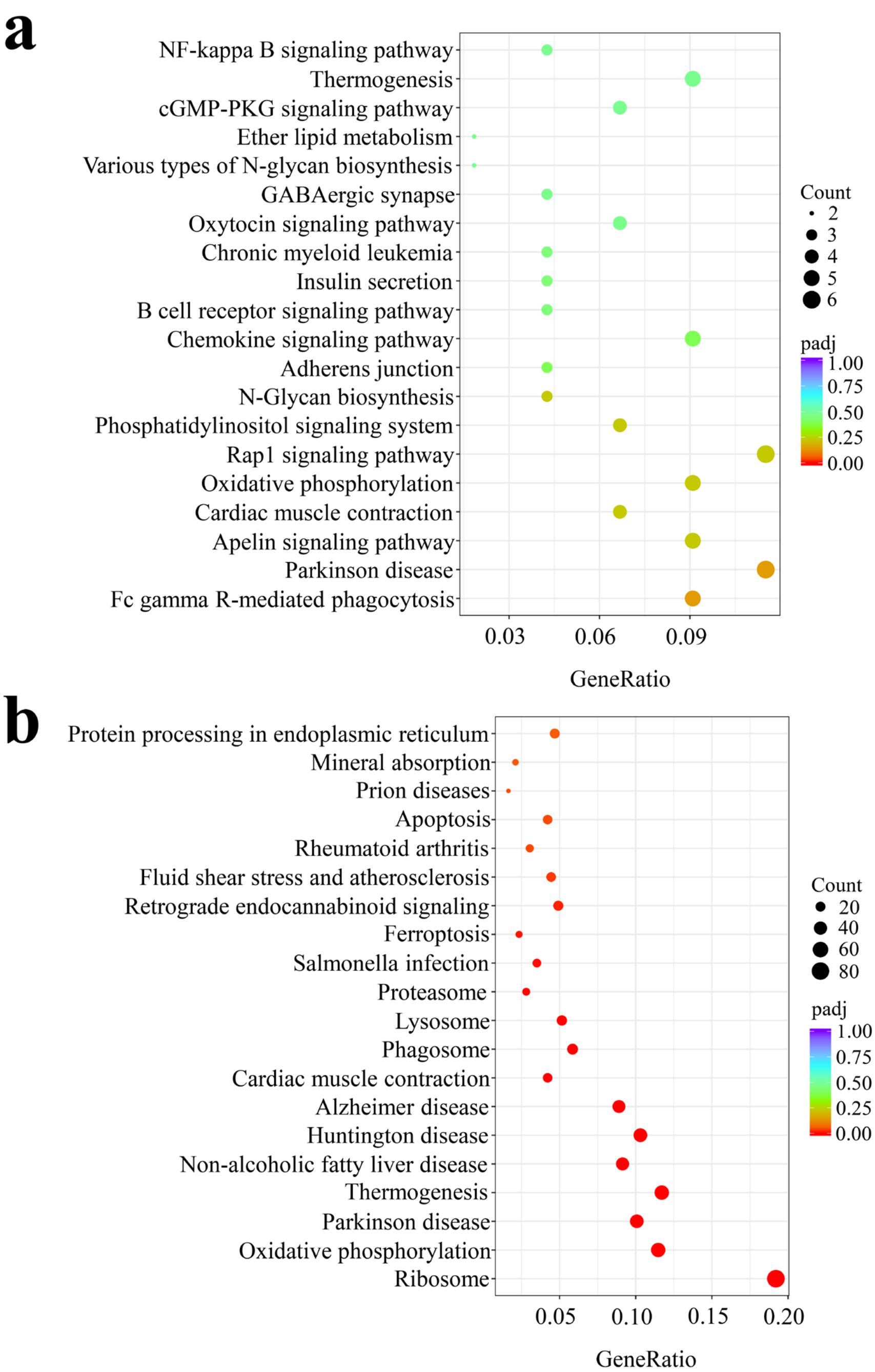

Supplementary Figure S18. KEGG functional enrichment analysis for genes that are up-regulated (a) or down-regulated (b) in microglia following *B. licheniformis* intervention. The horizontal axis (GeneRatio) represents the ratio of the number of DEGs enriched in a specific pathway to the total number of DEGs. The size of the dots is proportional to the number of genes (Count) involved in each pathway. The color gradient indicates the adjusted p-value (padj), with red representing higher statistical significance (lower padj values).
