# Supplementary material for: Bacillus Licheniformis‐Induced Cognitive Decline in Mice Linking Gut Microbiota Dysbiosis, Metabolic Perturbations, and Hippocampal Neuroinflammation
Source: Adv Sci (Weinh). 2026 Nov 9:e78686. Online ahead of print. doi: 10.1002/advs.78686 (PMC13748769; doi:10.1002/advs.78686)
Supplement: Supplementary file 2 [file ADVS-9999-e78686-s002.zip › advs78686-sup-00011-S10. Single-Cell RNA sequencing metrics and quality control statistics analyzed by Cell Ranger.docx]

Supplementary Table S10. Single-Cell RNA sequencing metrics and quality control statistics analyzed by Cell Ranger

| Sample Name | *C. butyricum* | *B. licheniformis* | Control |
| --- | --- | --- | --- |
| Estimated number of cells | 11,156 | 13,069 | 8,362 |
| Mean reads per cell | 33,781 | 31,484 | 41,170 |
| Median genes per cell | 2,783 | 2,456 | 2,832 |
| Number of reads | 376,863,351 | 411,461,617 | 344,263,679 |
| Valid barcodes | 96.2% | 96.5% | 95.9% |
| Sequencing saturation | 70.5% | 71.8% | 74.5% |
| Q30 bases in barcode | 97.4% | 97.1% | 97.4% |
| Q30 bases in RNA read | 96.0% | 95.9% | 96.2% |
| Q30 bases in UMI | 98.0% | 97.9% | 98.0% |
| Reads mapped to genome | 96.4% | 96.5% | 96.5% |
| Reads mapped confidently to genome | 94.9% | 95.0% | 94.9% |
| Reads mapped confidently to intergenic regions | 3.3% | 3.5% | 3.6% |
| Reads mapped confidently to intronic regions | 25.9% | 22.7% | 22.5% |
| Reads mapped confidently to exonic regions | 65.6% | 68.8% | 68.8% |
| Reads mapped confidently to transcriptome | 83.7% | 82.8% | 83.6% |
| Reads mapped antisense to gene | 7.5% | 8.3% | 7.4% |
| Fraction reads in cells | 93.6% | 92.2% | 94.6% |
| Total genes detected | 23,781 | 23,981 | 23,531 |
| Median UMI counts per cell | 6,833 | 5,642 | 7,220 |
