# Supplementary material for: Bacillus Licheniformis‐Induced Cognitive Decline in Mice Linking Gut Microbiota Dysbiosis, Metabolic Perturbations, and Hippocampal Neuroinflammation
Source: Adv Sci (Weinh). 2026 Nov 9:e78686. Online ahead of print. doi: 10.1002/advs.78686 (PMC13748769; doi:10.1002/advs.78686)
Supplement: Supplementary file 2 [file ADVS-9999-e78686-s002.zip › advs78686-sup-00012-S11. Markers of Major Cell Types in the Mouse Hippocampus.docx]

Supplementary Table S11. Markers of Major Cell Types in the Mouse Hippocampus

| **Cell Type** | **Marker Genes (Representative)** | **Key Notes and Subtype Markers** |
| --- | --- | --- |
| Oligodendrocytes | Mbp, Plp1, Mag, Mog, Olig2, Pdgfra | Pdgfra is often used to mark oligodendrocyte precursor cells. Olig2 is a key transcription factor and is commonly used as a pan-lineage marker. During maturation, genes such as Tns3, Mal, and Mog are expressed. |
| Microglia | C1qc, Iba1 (Aif1 gene), Tmem119, Cx3cr1 | Microglia are highly heterogeneous and exist in different functional states. For example, inflammation-associated microglia (IAM) highly express pro-inflammatory cytokine genes such as Tnf, Il1b, and Ccl2. Disease-associated microglia (DAM) may express genes like Clec7a and Trem2. |
| Astrocytes | Gfap, Aqp4, Slc1a2 (EAAT2) | Gfap and Aqp4 are markers for astrocytes. |
| Endothelial Cells | Flt1 (VEGFR1), Cldn5 (Claudin-5), Pecam1 (CD31) | Flt1 is a primary marker for endothelial cells, and Cldn5 and Pecam1 are also commonly used markers. |
| Excitatory Neurons | Slc17a6 (VGLUT2), Slc17a7 (VGLUT1) | Excitatory neurons typically express markers of glutamatergic neurons, such as the vesicular glutamate transporters Slc17a6 and Slc17a7. |
| Choroid Plexus Cells | Ttr (Transthyretin), Klk6 | Choroid plexus cells are primarily responsible for cerebrospinal fluid secretion. Ttr is a classic, specific marker for these cells. |
| Plasma Cells | Jchain, Sdc1 (CD138), Xbp1, Prdm1 (Blimp-1) | Plasma cells are terminally differentiated B cells responsible for antibody secretion. Their markers include Jchain, Sdc1, and the transcription factors Xbp1 and Prdm1. |
| B Cells | Cd19, Cd79a, Ms4a1 (CD20) | Markers for B cells include Cd19, Cd79a, etc. In a healthy central nervous system, the number of B cells is very low. |
| Pericytes | Pdgfrb, Cspg4 (NG2), Rgs5 | Pericytes are located on capillary walls. Pdgfrb and Cspg4 (NG2) are key markers. Note that Cspg4 is also expressed in oligodendrocyte precursor cells, requiring combination with other markers for distinction. |
