# Supplementary material for: Bacillus Licheniformis‐Induced Cognitive Decline in Mice Linking Gut Microbiota Dysbiosis, Metabolic Perturbations, and Hippocampal Neuroinflammation
Source: Adv Sci (Weinh). 2026 Nov 9:e78686. Online ahead of print. doi: 10.1002/advs.78686 (PMC13748769; doi:10.1002/advs.78686)
Supplement: Supplementary file 2 [file ADVS-9999-e78686-s002.zip › advs78686-sup-00016-S15. Probiotic strains used in this study.docx]

**Supplementary Table S15: Probiotic strains used in this study**

| **Species** | **Strain Designation** | **Manufacturer / Supplier** | **Catalog / Lot No.** |
| --- | --- | --- | --- |
| *Bacillus licheniformis* | ATCC14580 | Shanghai Microbial Preservation Co., Ltd | SHMCC D80320 |
| *Clostridium butyricum* | ATCC19398 | Shanghai Microbial Preservation Co., Ltd | SHMCC D82487 |
| *Bacillus cereus* | ATCC14579 | Shanghai Microbial Preservation Co., Ltd | SHMCC D80547 |
| *Lactobacillus acidophilus* | ATCC4356 | Shanghai Microbial Preservation Co., Ltd | SHMCC D80367 |
| *Bifidobacterium* | ATCC55813 | Shanghai Microbial Preservation Co., Ltd | SHMCC D81420 |
