# Supplementary material for: Bacillus Licheniformis‐Induced Cognitive Decline in Mice Linking Gut Microbiota Dysbiosis, Metabolic Perturbations, and Hippocampal Neuroinflammation
Source: Adv Sci (Weinh). 2026 Nov 9:e78686. Online ahead of print. doi: 10.1002/advs.78686 (PMC13748769; doi:10.1002/advs.78686)
Supplement: Supplementary file 2 [file ADVS-9999-e78686-s002.zip › advs78686-sup-0007-S6. Quality control metrics and filtering statistics for scRNA - sequencing samples.docx]

Supplementary Table S6. Quality control metrics and filtering statistics for scRNA - sequencing samples

| Sample Name | *C. butyricum* | *B. licheniformis* | Control |
| --- | --- | --- | --- |
| min cells | 3 | 3 | 3 |
| low thresholds nGene | 200 | 200 | 200 |
| high thresholds nGene | 6000 | 6000 | 6000 |
| high thresholds percent mito | 30 | 25 | 30 |
| high thresholds percent HB | 5 | 5 | 5 |
| gene number | 23781 | 23981 | 23531 |
| cell number | 11156 | 13069 | 8362 |
| nGene median | 2782 | 2456 | 2832 |
| gene number filtered | 21259 | 21553 | 20861 |
| cell number filtered | 10177 | 11712 | 7636 |
| nGene median filtered | 2807 | 2451 | 2869 |

(1) min cells: The minimum number of cells in which a gene must be expressed. Genes expressed in fewer than this number of cells are filtered out.

(2) low thresholds nGene: The lower threshold for the number of genes detected per cell. Cells with fewer detected genes than this value are filtered out.

(3) high thresholds nGene: The upper threshold for the number of genes detected per cell. Cells with more detected genes than this value are filtered out.

(4) high thresholds percent mito: The maximum threshold for the percentage of UMIs (Unique Molecular Identifiers) derived from mitochondrial genes. Cells with a higher percentage than this value are filtered out.

(5) high thresholds percent HB: The maximum threshold for the percentage of UMIs derived from hemoglobin (red blood cell) genes. Cells with a higher percentage than this value are filtered out.

(6) gene number: Number of genes before filtering.

(7) cell number: Number of cells before filtering.

(8) nGene median: Median number of genes detected per cell before filtering.

(9) gene number filtered: Number of genes after filtering.

(10) cell number filtered: Number of cells after filtering.

(11) nGene median filtered: Median number of genes detected per cell after filtering.
